# Physical activity, psychological resilience, and adolescent mobile phone addiction—a meta-analytic structural equation modeling study

**DOI:** 10.3389/fpsyg.2026.1914677

**Published:** 2026-10-02

**Authors:** Shizhong Li, Lihong Chen, Shaohua Liu

**Affiliations:** Hunan Mechanical & Electrical Polytechnic, Changsha, Hunan, China

**Keywords:** adolescent physical activity, MASEM, mediating effect, mobile phone addiction, psychological resilience

## Abstract

**Background and aims:**

With the widespread use of mobile internet, smartphone addiction among adolescents has become a major global public health concern.

**Methods:**

This study synthesizes empirical evidence to quantitatively examine the relationships among adolescent physical activity (PA), psychological resilience (PR), and mobile phone addiction (MPA), and to explore the mediating role of PR. Following PRISMA guidelines, a systematic search of five databases (PubMed, Web of Science, PsycINFO, SPORTDiscus, CNKI) yielded 58 relevant articles (63 correlation records across the three relationship pairs), comprising cross-sectional and longitudinal studies with a total sample of 98,946 adolescents and young adults. Traditional meta-analysis and MASEM were combined; a random-effects model pooled effect sizes, and a two-stage structural equation modeling approach tested mediation.

**Results:**

There was a significant negative correlation between PA and MPA (*r* = −0.225 [95% CI: −0.282 to −0.166]). PA was positively correlated with PR (*r* = 0.370 [95% CI: 0.250 to 0.479]). PR was negatively correlated with MPA (*r* = −0.362 [95% CI: −0.518 to −0.184]). Neither Egger’s nor Begg’s test indicated statistically significant small-study effects for any of the three pairwise relationships. MASEM analysis revealed that PR plays a substantial mediating role, with an indirect effect of −0.120, accounting for 53.3% of the total effect. Substantial heterogeneity was observed across all analyses (I2 > 96%), which was partially explained by educational level, study design and measurement instrument in moderator analyses.

**Conclusion:**

PA is associated with lower MPA overall (*r* = −0.225); in the mediation model the direct effect is reduced to borderline non-significance, with the association substantially (~53%) mediated by psychological resilience. The findings suggest that effective interventions for adolescents and young adults with mobile phone addiction should combine physical-activity promotion with psychological components. However, these recommendations should be considered in light of the predominantly observational evidence base, and future intervention studies are needed to establish causal relationships.

**Systematic review registration:**

https://www.crd.york.ac.uk/PROSPERO/view/CRD420261398773, identifier: CRD420261398773.

## 1 Introduction

Rapid digital technology development and the widespread adoption of mobile internet have made smartphones indispensable tools for daily life, study, and social interaction among adolescents. The 55th Statistical Report on China’s Internet Development, released by the China Internet Network Information Center (CNNIC) in December 2024, indicated that the number of internet users aged 10–19 in China reached 186 million, accounting for 16.7% of all netizens (CNNIC, 2025). The smartphone penetration rate among the adolescent demographic exceeds 98% (Fang et al., 2024). While smartphones provide adolescents with convenient access to information and rich social experiences, they also introduce a series of problems that cannot be ignored. Mobile phone addiction has become a global public health issue affecting the physical and mental development of adolescents. Recent survey data indicate that mobile phone addiction among Chinese adolescents is increasingly severe. Mei et al. (2022) conducted a cross-sectional survey of 946 Chinese college students using the Mobile Phone Addiction Index and reported a mobile phone addiction detection rate of 36.6%, with severe addiction significantly associated with a range of psychological, physical, and sleep-related health problems.

Mobile phone addiction can lead to difficulties in concentration for adolescents, declining academic performance, reduced sleep quality, and may also lead to emotional problems such as anxiety, depression, and loneliness, potentially increasing impulsive behaviors and the risk of social isolation. Prolonged mobile phone use leads to a significant reduction in adolescent physical activity, resulting in increasingly prevalent sedentary behavior, phone addiction, and unwillingness to participate in sports. This poses a great risk to the physical health of adolescents (Zhu et al., 2023). Furthermore, the lack of sufficient physical activity among Chinese adolescents is severe. According to the “Report on the Physical Fitness and Health Monitoring of Chinese Students,” less than 30% of primary and secondary school students achieve at least 1 h of moderate-to-vigorous physical activity daily. For university students, this proportion is even lower, with over 60% reporting low levels of physical activity (Yang et al., 2020).

Internationally, the prevalence of problematic smartphone use among adolescents and young adults is similarly alarming. Studies from India report that 39–45% of young adults exhibit problematic smartphone use patterns (Haripriya et al., 2019; Kumar et al., 2024). In Turkey, Numanoğlu-Akbaş et al. (2020) found that university students with low physical activity levels were significantly more likely to exhibit smartphone addiction. In South Korea, longitudinal evidence by Kim and Ahn (2023) demonstrated that baseline exercise time predicted subsequent smartphone dependency among middle school students. In Europe, Cocozza et al. (2020) examined the relationship between leisure activities, including physical activity, and psychological resilience among Italian residents, underscoring the need for adolescent-specific evidence. In the United States, Belaire et al. (2024) investigated the relationship between social–emotional learning, resilience, and physical activity in school-aged children. In Hong Kong, Ho et al. (2015) reported that physical activity was associated with better mental health through higher resilience in Chinese adolescents. These international studies collectively highlight the global relevance of the PA–resilience–MPA nexus.

The selection of psychological resilience as the principal mediator in the PA–MPA relationship is grounded in three complementary theoretical perspectives. First, Resilience Theory (Masten, 2001; Connor and Davidson, 2003) posits that resilience is not a fixed trait but a dynamic developmental process resulting from interactions between individuals and their environments. Physical activity, as a structured and challenging activity, provides repeated opportunities for adolescents to experience mastery, overcome obstacles, and build coping resources, thereby fostering resilience. Second, Self-Regulation Theory (Baumeister and Heatherton, 1996; Bandura, 1991) suggests that physical activity strengthens self-regulatory resources—such as goal-setting, self-monitoring, and impulse control—which are transferable to other domains including technology use. Individuals with stronger self-regulation are better equipped to manage their smartphone use and resist addictive patterns. Third, the Interaction of Person-Affect-Cognition-Execution (I-PACE) model of behavioral addiction (Brand et al., 2016) identifies psychological resilience as a key protective personal variable that reduces vulnerability to developing behavioral addictions, including problematic smartphone use. According to this model, individuals with lower resilience are more likely to use smartphones as a coping mechanism for stress and negative emotions, whereas those with higher resilience can employ adaptive coping strategies. Together, these three frameworks provide a coherent theoretical rationale for hypothesizing psychological resilience as a mediator of the PA–MPA relationship.

Despite these advances, both international and Chinese research share limitations. Most studies are cross-sectional (>85%), rely on Chinese or college-student samples (>70%), and test single mediators rather than integrated models. No study has used meta-analytic structural equation modeling (MASEM) to quantitatively synthesize the mediating role of psychological resilience across the PA-MPA relationship. This gap directly justifies the present MASEM study. Therefore, this study employs the MASEM method to comprehensively analyze and summarize the results of relevant domestic and international literature, exploring the relationships among adolescent physical activity, psychological resilience, and mobile phone addiction, and clarifying the extent of the mediating role of psychological resilience. This is significant for further understanding the mechanism underlying the impact of physical activity on adolescent mobile phone addiction and provides a reference for preventing and intervening in adolescent mobile phone addiction.

Based on meta-analysis and structural equation modeling, this study synthesizes relevant domestic and international literature, analyzes the relationships among adolescent physical activity, psychological resilience, and mobile phone addiction, and explores the degree and magnitude of the mediating effect of psychological resilience. The main objectives are: First, to use traditional meta-analysis to obtain the overall effect sizes for the relationships between adolescent physical activity and mobile phone addiction, physical activity and psychological resilience, and psychological resilience and mobile phone addiction, measuring the closeness of associations between the different variable pairs. Second, to compare differences among studies to identify sources of heterogeneity. Third, to examine whether publication bias affects the conclusions, ensuring the reliability of the findings. Fourth, to use the MASEM method to construct an SEM, test whether psychological resilience plays a mediating role, estimate the direct, indirect, and total effect sizes, and determine the proportion of the mediation effect.

Based on the gaps identified in the literature, the specific research questions are as follows: (1) Is there a significant negative correlation between adolescent physical activity and mobile phone addiction? (2) Is physical activity significantly associated with the higher level of psychological resilience in adolescents? (3) Does psychological resilience mediate the relationship between adolescent physical activity and mobile phone addiction? (4) If a mediating effect exists, what is its magnitude?

## 2 Methods

### 2.1 Study design

This study uses traditional meta-analysis and meta-analytic structural equation modeling (MASEM; Assink and Wibbelink, 2016). First, traditional meta-analysis is employed to synthesize relevant domestic and international literature, obtaining pooled effect sizes for the relationships among adolescents’ and young adults’ physical activity, psychological resilience, and mobile phone addiction. Second, based on the results of the meta-analysis, a structural equation model is constructed to examine the mediating role of psychological resilience in the relationship between adolescent physical activity and mobile phone addiction. This approach overcomes the instability of results from single studies due to small sample sizes, reflects the relationships among variables on a larger scale, and enhances the reliability and persuasiveness of the findings.

### 2.2 Conceptual definitions

The World Health Organization defines physical activity as any bodily movement produced by skeletal muscles that results in energy expenditure. Physical activity covers all forms of body movement in daily life, including occupational activities, housework, transportation, and leisure activities. Psychological resilience was initially used to describe an individual’s ability to recover well after experiencing severe setbacks. Early research considered resilience a fixed personality trait. However, it was later discovered that resilience is not static but a dynamic developmental process, resulting from continuous interaction between an individual and their environment. Mobile phone addiction, a type of behavioral addiction, refers to an individual’s inability to control their mobile phone use, leading to physical, mental, and social life problems.

### 2.3 Literature search and screening

This study searched 5 authoritative databases, both in Chinese and English. Chinese databases were CNKI (China National Knowledge Infrastructure). English databases included PubMed, Web of Science, PsycINFO and SPORTDiscus. The search period covered from January 2015 to May 2026(The choice of January 2015 as the cut-off point was primarily driven by the fact that smartphones became widely prevalent around this time). Chinese search terms included: physical activity (体力活动, 体育锻炼, 运动), psychological resilience (心理韧性, 心理弹性), mobile phone addiction (手机成瘾, 手机依赖, 智能手机成瘾, 问题性手机使用). English search terms included: physical activity, exercise, sport, resilience, psychological resilience, mobile phone addiction, smartphone addiction, problematic mobile phone use. The search used a combination of MeSH terms and free text words. Search terms for different variables were combined with “AND,” while different expressions for the same variable were combined with “OR.”

### 2.4 Inclusion criteria

Studies meeting the following criteria were included: (1) Empirical research methods, including cross-sectional and longitudinal cohort studies; (2) Study participants were adolescents and young adults, defined as samples with a mean/median age within the 10–24 year band (e.g., junior high, senior high, and college/university students). Studies whose samples were predominantly within this band were retained even if a small proportion of participants slightly exceeded 24 years; (3) Reported correlation coefficients between at least two of the three variables (adolescent physical activity, psychological resilience, mobile phone addiction), or statistical data (standardized regression coefficients, t-values, *F*-values) that could be converted into correlation coefficients; (4) Provided specific sample size; (5) Published as journal articles or master’s/doctoral theses.

### 2.5 Exclusion criteria

Studies were excluded if they met any of the following criteria: (1) Non-empirical research such as reviews, commentaries, theoretical discussions, or conference abstracts; (2) Duplicate publications (only the earliest or most complete version retained); (3) Studies involving participants outside adolescents and young adults range or special populations such as patients with clinical diseases, elderly individuals, or individuals with disabilities; (4) Unable to extract effective effect size data, and authors could not be contacted to provide supplementary data; (5) Written in languages other than Chinese or English.

### 2.6 Data extraction and coding

Data extraction and coding were independently performed by two trained researchers from the study group. They first pre-extracted data from three randomly selected articles to standardize the extraction and coding rules before proceeding with full-sample extraction. Disagreements were resolved through discussion. If consensus could not be reached, the study supervisor made the final decision. Extracted information included four dimensions: basic literature characteristics (first author, publication year, document type), participant characteristics (total sample size, mean age, gender ratio, educational level), measurement tool information (scale names for physical activity, psychological resilience, and mobile phone addiction), and effect size data (correlation coefficients between variables or other statistical indicators convertible to correlation coefficients). Inter-rater agreement for the independent double data extraction was substantial to excellent (Cohen’s kappa = 0.87 for categorical variables; intraclass correlation coefficient = 0.92 for continuous variables).

A standardized coding protocol was followed: For studies reporting multiple correlation coefficients of the same type for the same relationship, the average was used as the effect size for that study, calculated using Fisher’s z-transformation to ensure appropriate weighting. If only standardized regression coefficients (β) were available, they were converted to Pearson correlation coefficients (r) using the formula *r* = 0.98β + 0.05λ (where λ = 1 when β is positive and λ = −1 when β is negative), as validated by Peterson and Brown (2005) through Monte Carlo simulations with a mean error of less than 0.01. This conversion approach has been widely used in meta-analytic research (Aloe and Becker, 2012). When both r and β were reported, the correlation coefficient was used directly. For cross-sectional studies with multiple measurement time points, baseline data were used for calculations to ensure comparability across studies.

### 2.7 Data analysis methods

#### 2.7.1 Traditional meta-analysis

Following the Hunter and Schmidt (1990) framework, the meta-analysis was conducted using Stata 19 software, with effect sizes calculated via Fisher’s z-transformation. Because the sampling distribution of the correlation coefficient r is not normal, all correlation coefficients were converted to Fisher’s Z scores for pooling effect sizes, and then converted back to r (Briki, 2018). Given expected differences in sample characteristics, scales used, and research methods across included studies, a random-effects model was chosen for pooling effect sizes. This model accounts for both within-study sampling variability and between-study differences, offering greater generalizability. Statistical significance of the pooled effect size was determined using the 95% confidence interval (CI); if the CI did not contain zero, the result was considered statistically significant.

Heterogeneity between studies was assessed using the Q-test and I^2^ statistic. A Q-test *p*-value < 0.05 indicated significant heterogeneity. I^2^ values: <25% suggested no significant heterogeneity, 25% ≤ I^2^ < 75% suggested moderate heterogeneity, and ≥75% suggested substantial heterogeneity. Publication bias was assessed using funnel plots, Egger’s and Begg’s linear regression test, judging by funnel plot symmetry and whether the Egger’s test *p*-value > 0.05 (Doucouliagos et al., 2014). If significant publication bias was detected, the trim-and-fill method was used to adjust the pooled effect size. Sensitivity analysis was performed using the leave-one-out method (iteratively removing one study and recalculating the pooled effect size) to evaluate the stability and reliability of the overall results.

Given the extremely high heterogeneity observed (I^2^ > 96% for all three analyses), moderator analyses were conducted to explore potential sources of between-study variability. Categorical moderators—including country/region (China vs. other countries), educational level (junior high school, senior high school, college/university), study design (cross-sectional vs. longitudinal), measurement instrument type—were examined using subgroup Q-tests. Continuous moderators (mean age, publication year, % male, study methodological quality) were examined using random-effects meta-regression.

#### 2.7.2 Meta-analytic structural equation modeling

This study used the R package metaSEM (version 1.3.0) following Cheung’s two-stage approach (Cheung, 2014). Stage 1: A random-effects model (metaSEM) was used to synthesize a pooled 3 × 3 correlation matrix. The three pairwise correlations were estimated from partly non-overlapping sets of studies: PA–MPA (k = 42), PA–PR (k = 13), and PR–MPA (k = 8). Of the 58 included studies, only two (Shen and Gao, 2024; Zhang and Gao, 2023) reported all three pairwise correlations, one reported two pairs (PA–PR and PR–MPA), and 55 reported a single pair (40 PA–MPA, 10 PA–PR, 5 PR–MPA). Each study contributed whichever correlations it reported; missing correlations were handled by full-information maximum-likelihood (FIML) estimation under a missing-at-random (MAR) assumption, implemented in metaSEM:tssem1. Because the three pairwise correlations were estimated from partly non-overlapping study sets, tssem1 jointly estimates the 3 × 3 pooled correlation matrix and its asymptotic covariance matrix under a random-effects model (REML). The pooled matrix was estimated from all 58 studies, with 1.0 on the diagonal and the REML/Fisher-z pooled correlations on the off-diagonals (positive-definiteness verified).

Stage 2: Based on the synthesized correlation matrix, a mediation model was constructed. The significance of the mediation effect was tested using the Bootstrap method (5,000 resamples), decomposing total, direct, and indirect effects. The specified mediation model was a saturated model (df = 0), implying perfect fit. Because the Stage-2 model is saturated (df = 0), conventional fit indices (χ^2^/df, RMSEA, CFI, TLI) are not informative and are reported only to document that the model exactly reproduces the pooled correlation matrix; conclusions rest on the path coefficients, their standard errors, 95% CIs, and the bootstrap CI of the indirect effect. In Stage 2, the asymptotic covariance matrix of the Stage-1 pooled correlations was supplied to metaSEM:tssem2 as the sampling covariance matrix, so that Stage-1 estimation uncertainty is propagated into the standard errors and bootstrap CIs of the Stage-2 path coefficients and the indirect effect. The Stage-1 output is the pooled 3 × 3 correlation matrix and its asymptotic covariance matrix (ACov), whose off-diagonal elements are non-zero and reflect the shared information from the three studies reporting multiple correlation pairs. The full ACov was passed to metaSEM:tssem2, so Stage-1 estimation uncertainty is propagated into Stage-2 standard errors and the bootstrap CI of the indirect effect. Consequently, the Stage-2 total effect equals the pooled PA–MPA correlation. Additional analysis details, including the study-level matrices and full MASEM code, are available in the Supplementary Table S8.

### 2.8 Quality assessment

The methodological quality of included studies was assessed using the Newcastle-Ottawa Scale (NOS), adapted for cross-sectional and longitudinal observational studies (Wells et al., 2009). The methodological quality was assessed using the 9-item (NOS; Wells et al., 2009), covering three domains: Selection, Comparability, and Outcome (maximum = 9 points). Given the large proportion of cross-sectional work, we applied established cross-sectional adaptations of cohort-specific NOS items, using the same nine items across all studies to generate comparable total scores. Each item was scored 1 (criterion explicitly met) or 0 (unmet/unreported); no points were awarded through inference to ensure conservative ratings. Total scores were categorized as high (7–9), moderate (4–6), and low (0–3) quality. Two researchers independently completed quality appraisal from full-texts, resolving disagreements via discussion. Item-level ratings for all 58 studies are provided in Supplementary Table S7.

## 3 Results

### 3.1 Literature search results

Study selection was performed following PRISMA 2020 guidelines. After removal of duplicates, bibliographic records underwent title-and-abstract screening. Potentially relevant citations proceeded to full-text report assessment. Full-text reports were excluded for four predefined reasons: (1) participants did not meet the target age or population criteria; (2) effect-size data could not be extracted; (3) non-empirical review publications; (4) absence of available quantitative effect-size estimates. No trial registries were searched for this systematic review. The full study-selection workflow is presented in Figure 1. Systematic searches across the 5 databases (Web of Science, PubMed, SPORTDiscus, CNKI, PsycINFO) initially yielded 1,368 records. After removing 286 duplicates using EndNote X9 software, 1,082 records remained for title and abstract screening. Screening titles and abstracts excluded 929 records (unrelated topic, non-empirical research), leaving 153 records for full-text eligibility assessment. During full-text assessment, 95 records were excluded. Finally, 58 studies with 63 correlation records met the inclusion criteria, comprising 54 cross-sectional and 4 longitudinal/cross-lagged studies, with a total sample covering 98,946 adolescents and young adults.

**Figure 1:**
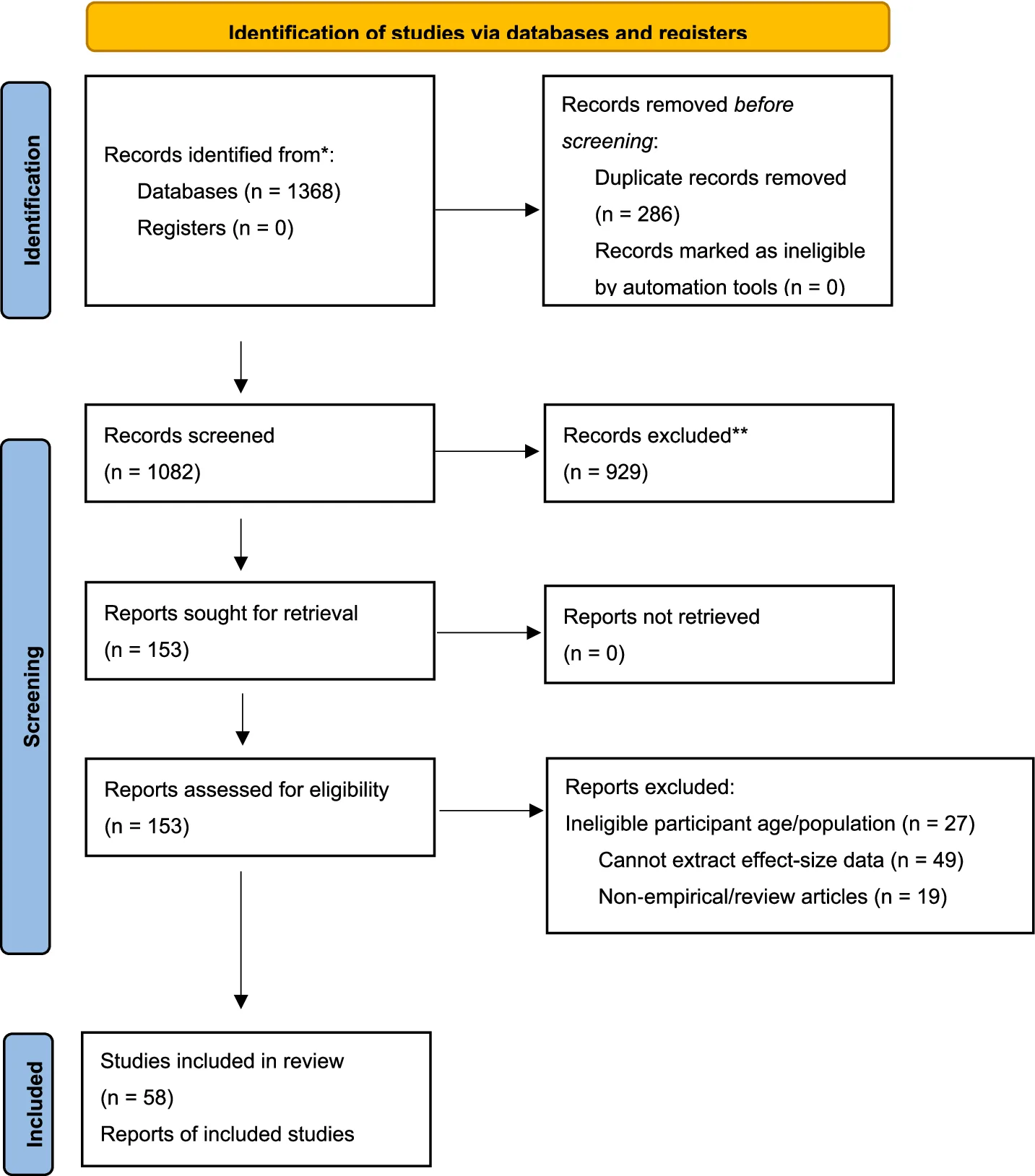
PRISMA flow diagram.

This meta-analysis included the following studies: Belaire et al. (2024); Cao et al. (2023); Cetin et al. (2022); Ceylan and DemİRdel (2023); Chao et al. (2022); Chen and Huan (2021); Chen et al. (2022); Cui and Zhang (2022); Ding et al. (2021); Dong Yaqi (2023); Gao et al. (2023); Gong et al. (2023); Guo et al. (2022); Han et al. (2023); Haripriya et al. (2019); Ho et al. (2015); Hu et al. (2024); Jin et al. (2024); Huang et al. (2022); Kim et al. (2015); Kim and Ahn (2023); Kumar et al. (2024); Li et al. (2021); Li et al. (2022); Li et al. (2023); Lin et al. (2022); Liu (2020); Liu and Sun (2023); Lu et al. (2022); Ma et al. (2022); Meng et al. (2024); Niu (2023); Numanoğlu-Akbaş et al. (2020); Sezer Efe et al. (2023); Shen and Gao (2024); Su et al. (2024); Tian et al. (2025); Tong et al. (2022); Tong and Meng (2023); Wan and Ren (2023); Wang et al. (2023); Wang et al. (2024); Wei (2023); Wu et al. (2024); Xiao (2022); Xie et al. (2023); Xu et al. (2023); Yang et al. (2019); Zeng et al. (2022); Zhang D. et al. (2022); Zhang Z. et al. (2022); Zhang and Gao (2023); Zhang et al. (2023); Zhao et al. (2022); Zhao et al. (2024); Zheng and Ma (2020); Zhou and zhou (2022); Zhu et al. (2023). The Summary of characteristics of studies included in the meta-analysis is provided in the Supplementary Table S6. Item-level NOS ratings for all 58 studies are reported in Supplementary Table S7. The mean total score was 6.50 (SD = 1.22; median 6.5, range 3–9). Twenty-nine studies (50.0%) were rated high quality (7–9), 28 (48.3%) moderate quality (4–6) and one (1.7%) low quality (0–3). All studies met the two comparability items and the outcome-assessment item, and 81.0% reported an effect size with its confidence interval or with sufficient statistics for its computation. Deductions were concentrated in three items: sampling representativeness (met by 41.4%), the reporting of a response or valid-questionnaire rate (41.4%), and documentation of the temporal structure of data collection (39.7%), reflecting the predominance of convenience samples in this literature. Mean scores were comparable across relationship pairs (PA–MPA 6.62, PA–PR 6.38, PR–MPA 5.38).

### 3.2 Meta-analysis results

A random-effects model was used to perform meta-analyses on the three pairwise relationships. The pooled effect sizes are summarized in Table 1.

**Table 1:** Random-effects univariate meta-analysis results.

Variable relationship	k	n	Pooled correlation r	95% CI	*p*-value	I^2^ (%)
PA–MPA	42	70,027	−0.225	[−0.282, −0.166]	<0.001	98.46
PA–PR	13	13,100	0.370	[+0.250, +0.479]	<0.001	98.40
PR–MPA	8	18,800	−0.362	[−0.518, −0.184]	<0.001	99.44

k, number of studies; n, number of total sample size; I^2^, heterogeneity index. PA, Physical Activity; PR, Psychological Resilience; MPA, Mobile Phone Addiction.

#### 3.2.1 Pooled effect size for physical activity and Mobile phone addiction

A traditional meta-analysis was performed on 42 effect sizes from 42 studies, encompassing a total sample of 70,027 adolescents, using a random-effects model. The results showed a significant negative correlation between adolescent physical activity and mobile phone addiction, with a pooled correlation coefficient *r* = −0.225 (95% CI: [−0.282, −0.166]), Z = −7.24, *p* < 0.001. According to Cohen’s guidelines, this effect size is considered small-to-medium. A forest plot displaying individual study effect sizes, their 95% CIs, and the pooled result is presented in Figure 2.

**Figure 2:**
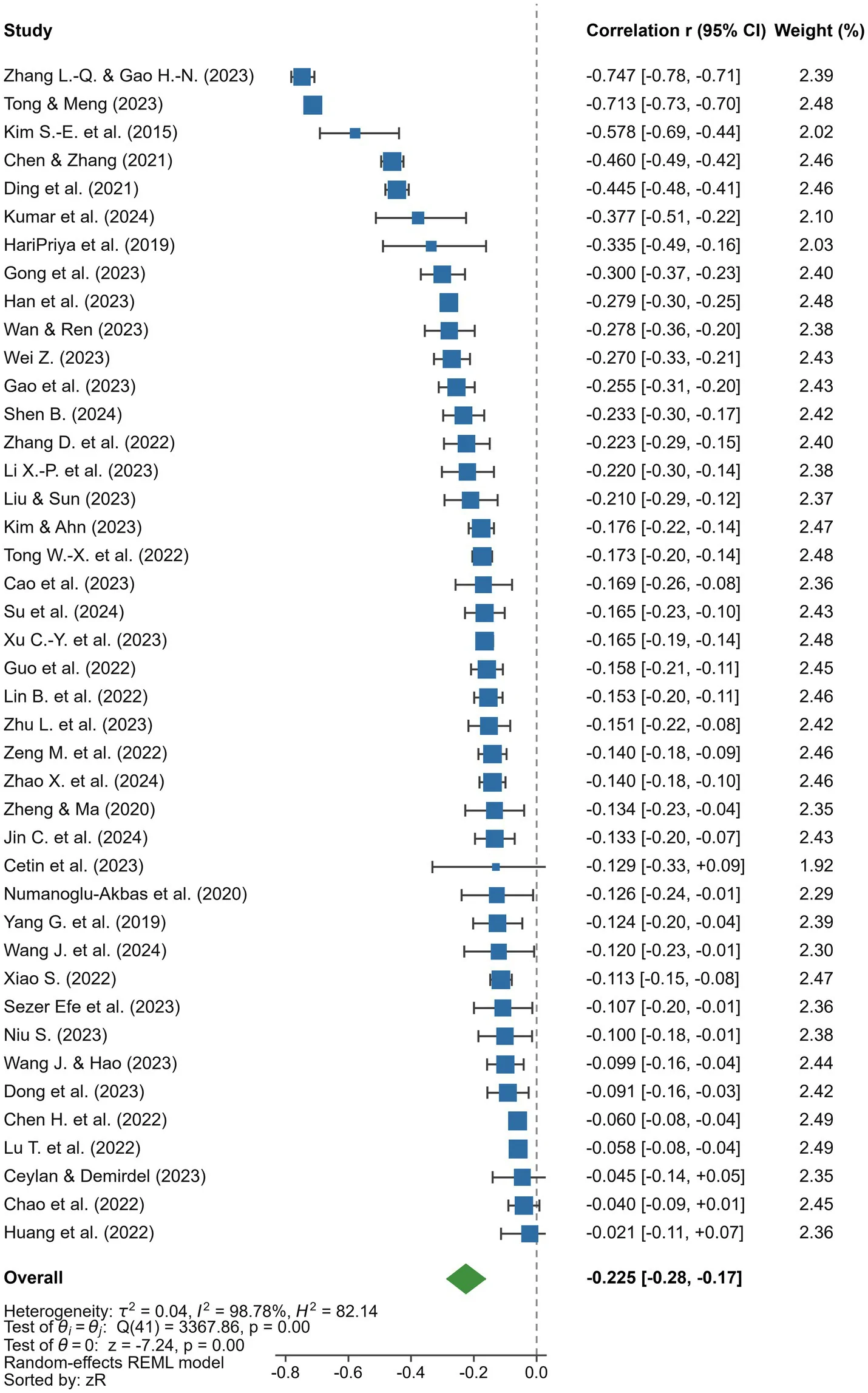
Forest plot for physical activity and mobile phone addiction.

#### 3.2.2 Pooled effect size for physical activity and psychological resilience

A traditional meta-analysis was performed on 13 effect sizes from 13 studies, encompassing a total sample of 13,100 adolescents, using a random-effects model. The results showed a significant positive correlation between adolescent physical activity and psychological resilience, with a pooled correlation coefficient *r* = 0.370 (95% CI: [0.250, 0.479]), Z = 5.50, *p* < 0.001. According to Cohen’s guidelines, this effect size is considered medium. A forest plot is presented in Figure 3.

**Figure 3:**
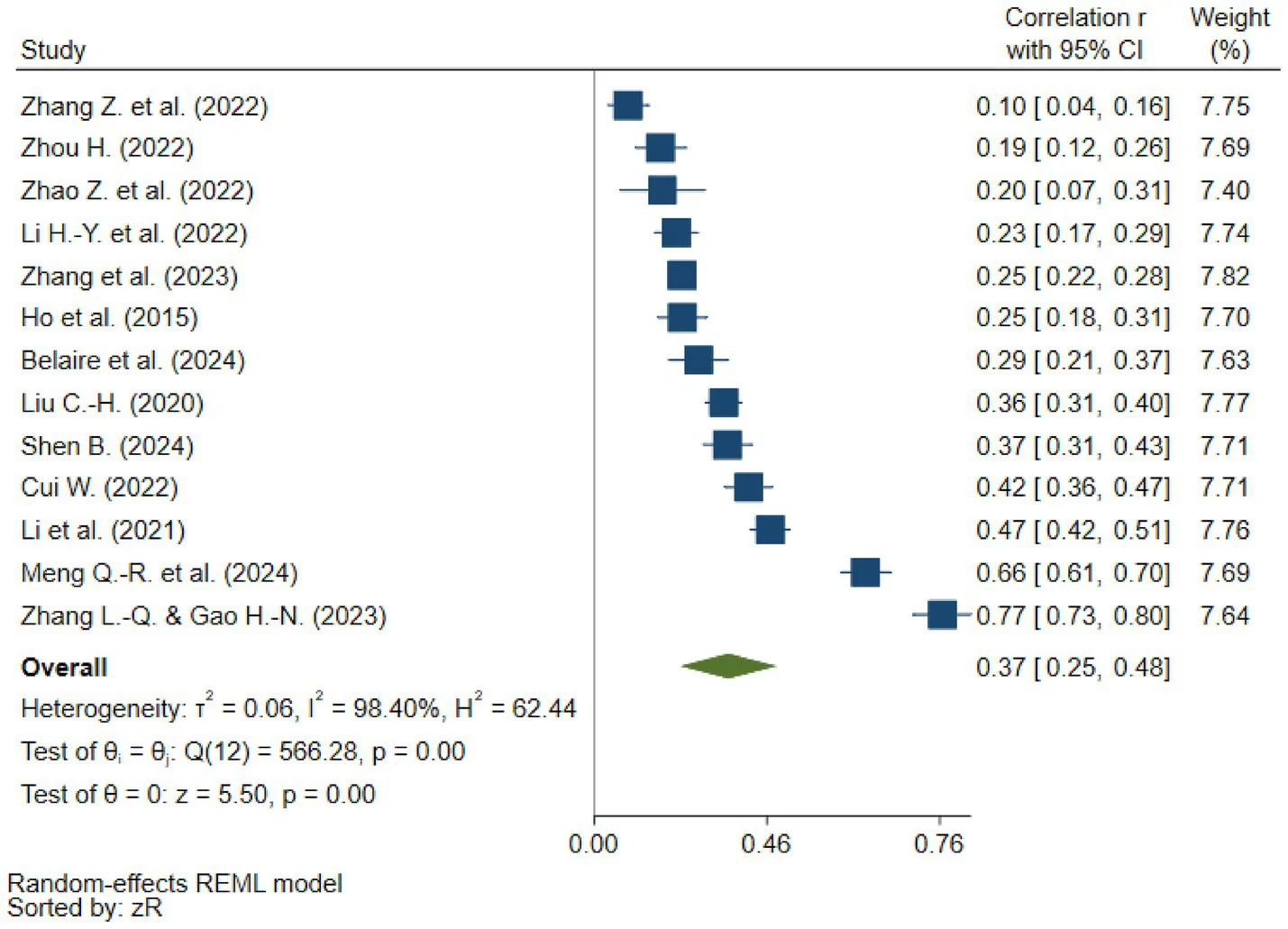
Forest plot for physical activity and psychological resilience.

#### 3.2.3 Pooled effect size for psychological resilience and Mobile phone addiction

A traditional meta-analysis was performed on 8 effect sizes from 8 studies, encompassing a total sample of 18,800 adolescents, using a random-effects model. The results showed a significant negative correlation between adolescent psychological resilience and mobile phone addiction, with a pooled correlation *r* = −0.362 (95% CI: [−0.518, −0.184]), Z = −3.60, *p* < 0.001. According to Cohen’s guidelines, this effect size is considered medium. A forest plot is presented in Figure 4.

**Figure 4:**
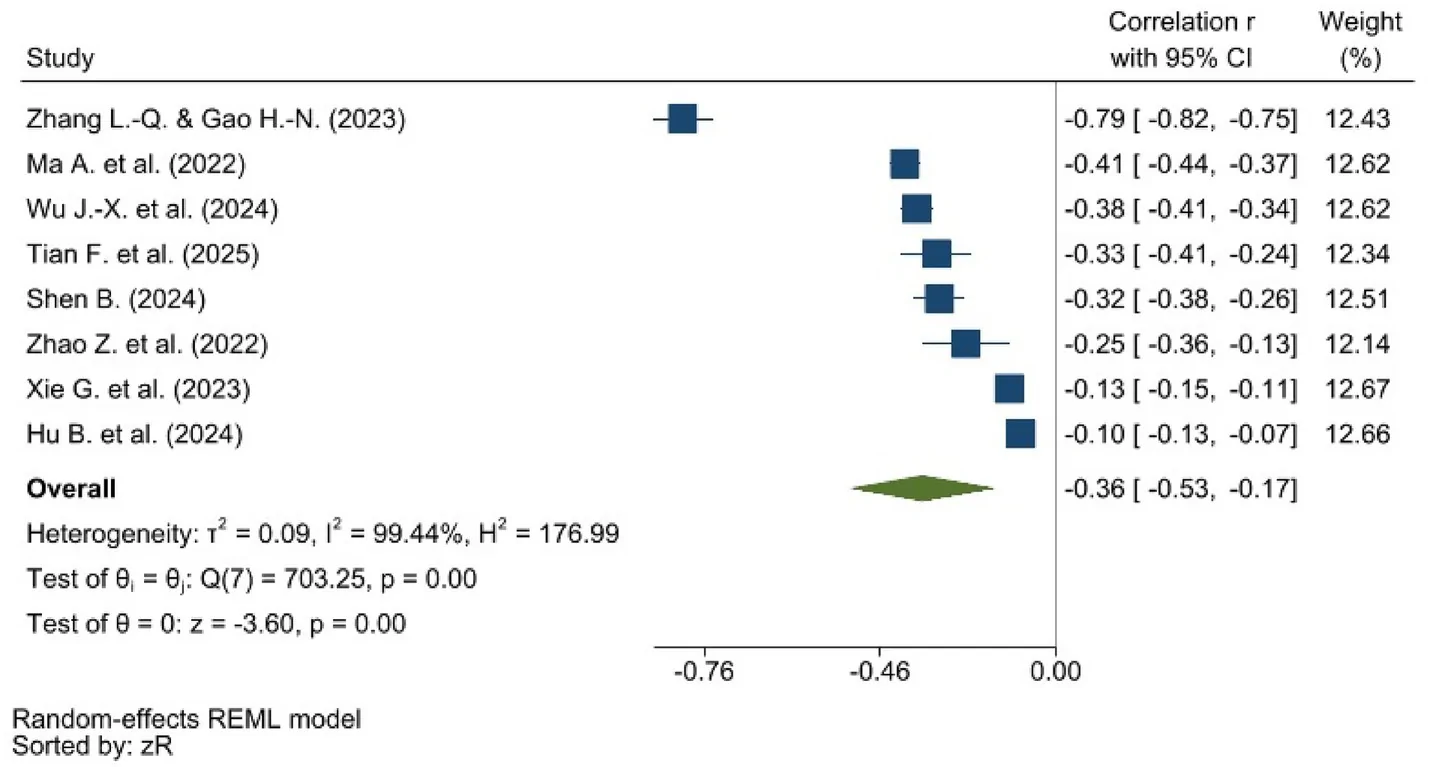
Forest plot for psychological resilience and mobile phone addiction.

#### 3.2.4 Publication bias test results

Publication bias was assessed using Egger’s linear regression test and Begg’s rank-correlation test, supplemented by visual inspection of funnel plots (Supplementary Figures S1–S3). For the PA–MPA relationship (k = 42), Egger’s test yielded an intercept of −1.04 (SE = 1.468, *p* = 0.478) and Begg’s test was non-significant (z = −1.82, *p* = 0.072); the funnel plot appeared approximately symmetric. For the PA–PR relationship (k = 13), Egger’s intercept was 1.83 (SE = 7.285, *p* = 0.801) and Begg’s test was non-significant (z = 0.79, *p* = 0.428); the funnel plot was symmetric. For the PR–MPA relationship (k = 8), Egger’s intercept was −5.06 (SE = 6.316, *p* = 0.423) and Begg’s test was non-significant (z = −0.37, *p* = 0.902); broadly symmetric, although the small number of studies limits visual assessment. Overall, neither Egger’s nor Begg’s test indicated statistically significant small-study effects for any of the three pairwise relationships, suggesting that publication bias is unlikely to have substantially distorted the pooled estimates.

#### 3.2.5 Sensitivity analysis results

A leave-one-out sensitivity analysis was performed to examine the influence of any single study on the pooled estimates (Supplementary Figures S4–S6). For each of the three relationships, sequential removal of any individual study yielded pooled correlations that remained stable around the overall estimates. No single study substantially altered the pooled estimates. These results indicate that the overall findings are robust and not unduly driven by any single study. One effect (Zhao et al., 2022, PA–PR, *r* = 0.195) was taken from an unadjusted, single-predictor standardized regression coefficient (β) rather than a zero-order correlation; no effect was converted from an *adjusted* standardized regression coefficient. Excluding this study in a sensitivity analysis left all results essentially unchanged.

#### 3.2.6 Subgroup and Meta-regression analysis

Subgroup analyses were conducted to explore sources of heterogeneity (Table 2). For the PA–MPA relationship, a significant between-group difference was detected only for study design (Qb = 8.23, *p* = 0.004); the negative PA–MPA association was stronger in cross-sectional studies (*r* = −0.237, k = 38) than in longitudinal/cross-lagged studies (*r* = −0.120, k = 4). The between-group tests for region (Qb = 0.03, *p* = 0.874), education level (Qb = 1.14, *p* = 0.567), and physical-activity measurement instrument (Qb = 3.10, *p* = 0.212) were not significant, indicating that these factors did not explain heterogeneity in the PA–MPA association. For the PA–PR relationship, a significant subgroup difference emerged for the PA measurement instrument (Qb = 6.21, *p* = 0.013); the PA–PR association was stronger when PA was assessed with PARS-3 (*r* = 0.453, k = 8) than with other/self-report instruments (*r* = 0.251, k = 4). The test for education level was not significant (Qb = 4.62, *p* = 0.099), and no between-group comparison was available for region, because only one multistudy subgroup (Chinese mainland) was estimable. For the PR–MPA relationship, subgroup differences were significant for education level (Qb = 5.56, *p* = 0.018); the association was stronger (more negative) in Secondary (12–18 y) (*r* = −0.505, k = 4) than in tertiary samples (*r* = −0.194, k = 4). No between-group comparison was available for region or study design, because all included PR–MPA studies were cross-sectional and conducted in Chinese mainland.

**Table 2:** Subgroup analyses of the three pairwise relationships.

Moderator	Subgroup	PA–MPA	PA–PR	MPA–PR
Region	Chinese mainland & regions	−0.222 [−0.287, −0.156] (34)	0.376 [0.247, 0.492] (12)	−0.362 [−0.490, −0.219] (8)
	Other countries	−0.233 [−0.348, −0.110] (8)	0.294 (1)	—
	*Q_b_ test*	*Qb = 0.03, p = 0.874*	*–*	*–*
Education level	Mixed	−0.223 (1)	—	—
	Primary/Junior (<12 y)	−0.177 [−0.265, −0.086] (3)	0.251 [0.224, 0.278] (3)	—
	Secondary (12–18 y)	−0.334 [−0.602, 0.002] (4)	0.559 [0.109, 0.819] (2)	−0.505 [−0.687, −0.264] (4)
	Tertiary (18–24 y)	−0.215 [−0.272, −0.156] (34)	0.359 [0.230, 0.475] (8)	−0.194 [−0.282, −0.102] (4)
	*Q_b_ test*	*Qb = 1.14, p = 0.567*	*Qb = 4.62, p = 0.099*	*Qb = 5.56, p = 0.018*
Study design	Cross-sectional	−0.237 [−0.298, −0.174] (38)	0.370 [0.262, 0.469] (13)	−0.362 [−0.490, −0.219] (8)
	Longitudinal/Cross-lagged	−0.120 [−0.169, −0.070] (4)	—	—
	*Q_b_ test*	*Qb = 8.23, p = 0.004*	*–*	*–*
PA instrument	PARS-3	−0.267 [−0.352, −0.177] (22)	0.453 [0.298, 0.585] (8)	—
	IPAQ	−0.169 [−0.250, −0.086] (12)	0.098 (1)	—
	Other/Self-report	−0.172 [−0.261, −0.080] (8)	0.251 [0.226, 0.275] (4)	−0.362 [−0.490, −0.219] (8)
	*Q_b_ test*	*Qb = 3.10, p = 0.212*	*Qb = 6.21, p = 0.013*	*–*

Cell entries are pooled correlations r [95% CI] (k = number of independent studies). Qb rows report the between-group heterogeneity test for that moderator within each relationship. — = not estimable (only one subgroup contributed data, or no study available). PA, physical activity; MPA, mobile phone addiction; PR, psychological resilience; PARS-3, Physical Activity Rating Scale-3; IPAQ, International Physical Activity Questionnaire.

Meta-regression analyses (Table 3) showed that the proportion of male participants significantly moderated the PA–MPA association (b = −0.007, SE = 0.003, *p* = 0.023; R^2^ = 10.3%). In contrast, methodological quality (nine-item NOS total), mean age, and publication year did not significantly moderate any of the three relationships (all *p* > 0.05).

**Table 3:** Meta-regression result.

Moderator	PA-MPA	PA-PR	MPA-PR
Mean age	k = 33 b = 0.00774 (SE = 0.01480) *p* = 0.605 R^2^ = 0.0%	k = 7 b = 0.01413 (SE = 0.03264) *p* = 0.683 R^2^ = 0.0%	k = 5 b = 0.02535 (SE = 0.11079) *p* = 0.834 R^2^ = 0.0%
% male	k = 33 b = −0.00694 (SE = 0.00289) *p* = 0.023 R^2^ = 10.3%	k = 8 b = 0.00943 (SE = 0.01314) *p* = 0.500 R^2^ = 0.0%	k = 8 b = −0.00546 (SE = 0.00768) *p* = 0.503 R^2^ = 0.0%
Publication year	k = 42 b = 0.02291 (SE = 0.01980) *p* = 0.254 R^2^ = 0.0%	k = 13 b = 0.02857 (SE = 0.03123) *p* = 0.380 R^2^ = 0.0%	k = 8 b = 0.04157 (SE = 0.11412) *p* = 0.728 R^2^ = 0.0%
NOS quality score (9-item total)	k = 42, b = 0.00335 (SE = 0.02870), *p* = 0.908, R^2^ = 0.0%	k = 13, b = 0.05341 (SE = 0.05109), *p* = 0.318, R^2^ = 0.7%	k = 8, b = −0.08857 (SE = 0.09668), *p* = 0.395, R^2^ = 0.0%

Each cell reports the number of studies (k), unstandardized coefficient b with its standard error (SE), the *p* value, and the proportion of heterogeneity explained (R^2^). t, τ^2^(null), and τ^2^(residual) were omitted for brevity; available on request. PA, physical activity; MPA, mobile phone addiction; PR, psychological resilience.

### 3.3 MASEM mediation model test results

This study employed the two-stage MASEM procedure (Cheung, 2014). In Stage 1, a pooled 3 × 3 correlation matrix for physical activity (PA), psychological resilience (PR), and mobile phone addiction (MPA) was synthesized (Figure 5). In Stage 2, this matrix was used to fit a path model in which PR was specified as the mediator of the PA–MPA association. In this model, the path coefficients are standardized partial regression coefficients, so that the coefficient of a single-predictor path (PA → PR) equals the corresponding pooled correlation, whereas the coefficients entering MPA (PA → MPA direct, PR → MPA) are partial effects controlling for the other predictor. The total, direct, and indirect effects were decomposed using the standard identity total = direct + (a × b), and the proportion mediated was computed accordingly. Model specification and results are reported in Figure 6 and Table 4.

**Figure 5:**
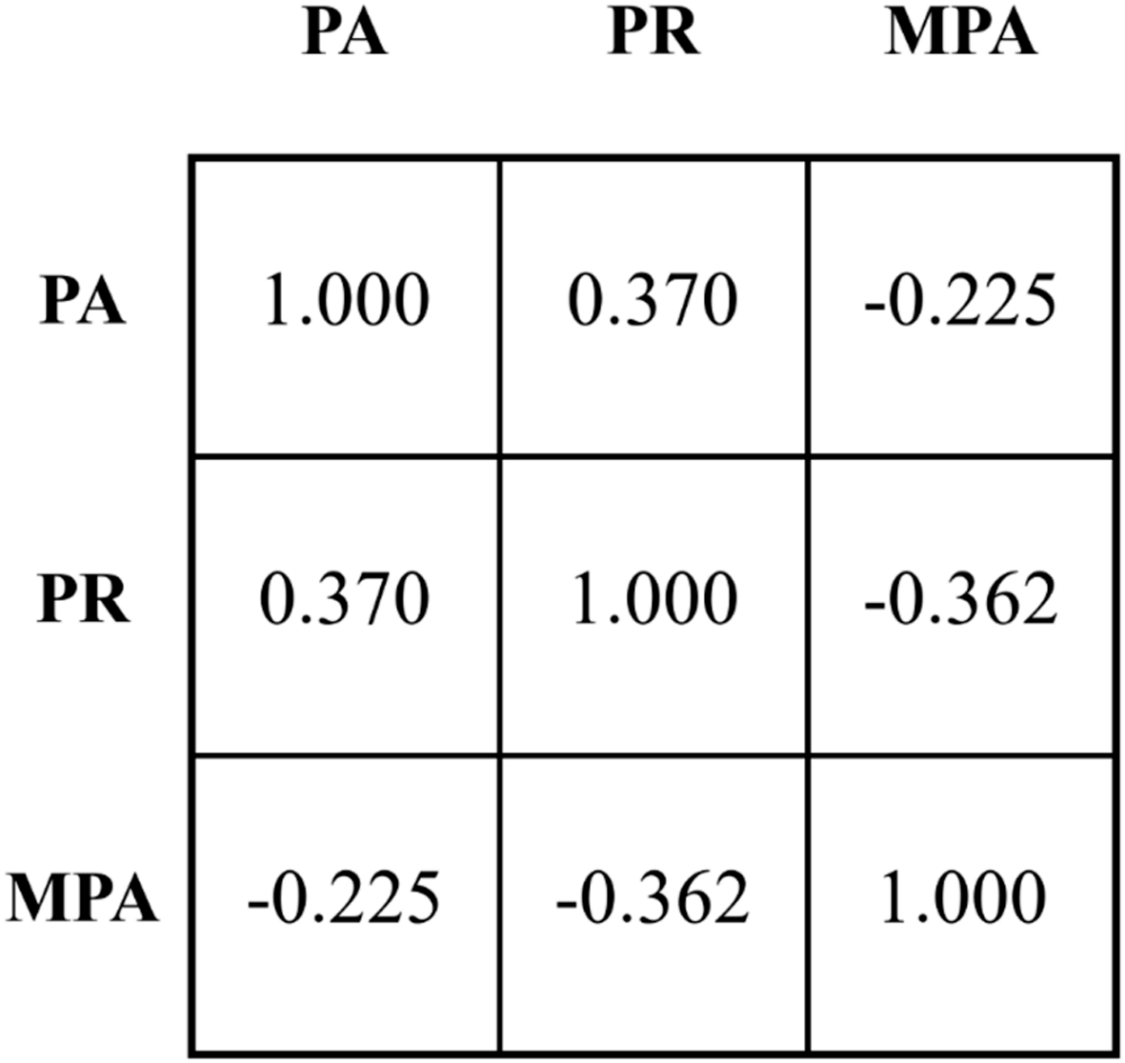
The pooled correlation r matrix.

**Figure 6:**
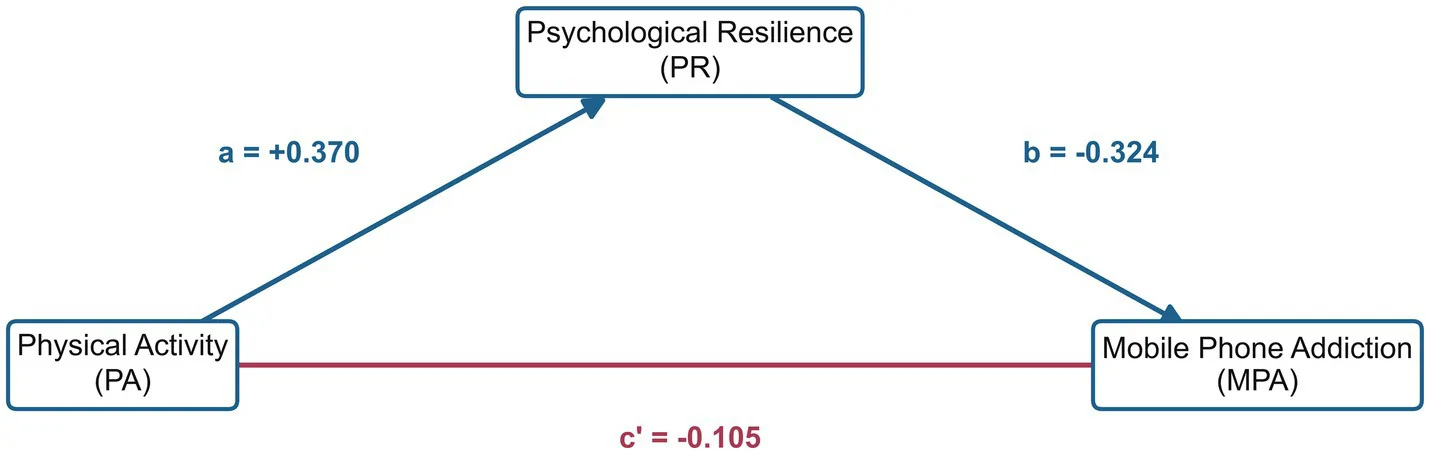
Meta-analytic structural equation model results.

**Table 4:** Standardized path coefficients and effect decomposition for the mediation model.

Path / Effect	Pooled r	Std. β	Std. error	*p*-value	95% CI
Direct paths					
PA → PR (a)	0.37	0.370	0.059	<0.001	[0.250, 0.479]
PR → MPA (b)	−0.362	−0.324	0.101	<0.001	[−0.509, −0.115]
PA → MPA (c′ direct)	−0.225	−0.105	0.055	0.053	[−0.208, +0.007]
Indirect effect					
PA → PR → MPA(a × b)		−0.120	0.043	0.006	[−0.209, −0.039]
Total effect					
PA → MPA (c total)		−0.225	0.030	<0.001	[−0.282, −0.166]

Proportion of total effect mediated = 53.3%. PA, Physical Activity; PR, Psychological Resilience; MPA, Mobile Phone Addiction.

Path analysis (Table 4) indicated that physical activity was negatively associated with mobile phone addiction in the SEM model (*β* = −0.105, *p* = 0.053), a marginally non-significant direct effect. Physical activity also had a significant positive effect on psychological resilience (*β* = 0.370, *p* < 0.001), and psychological resilience had a significant negative effect on mobile phone addiction (*β* = −0.324, *p* < 0.001). Therefore, psychological resilience played a significant mediating role, with an indirect effect size of −0.120, accounting for 53.3% of the total effect. This supports the hypothesis: physical activity is not directly associated with lower mobile phone addiction but is indirectly associated with lower mobile phone addiction by enhancing psychological resilience. Moreover, the mediation proportion is substantial (53.3%), indicating that psychological resilience accounts for more than half of the total effect, with other pathways still operating. The effect of physical activity on mobile phone addiction is more complex and likely involves other mediating variables such as self-control, loneliness, sleep quality, and peer relationships, which require further research. So this result supports the Structural Equation Model (see Figure 6) for “physical activity → psychological resilience → mobile phone addiction” hypothesis, enriches the understanding of the mechanisms linking adolescent physical activity to mental health, and provides a foundation for developing effective interventions.

## 4 Discussion

### 4.1 Interpretation of core findings

#### 4.1.1 Direct relationship between physical activity and mobile phone addiction

This study’s traditional meta-analysis of 58 studies (*N* = 98,946) revealed a significant negative correlation between adolescent physical activity and mobile phone addiction (*r* = −0.225, 95% CI: −0.282 to −0.166), a small-to-medium effect size. This finding, supported by numerous individual studies, suggests a robust negative association of physical activity against adolescent mobile phone addiction. This finding is consistent with previous studies or meta-analytic evidence. Zeng et al. (2022) reported a summary correlation of *r* = −0.21 for Chinese college students, and Lin et al. (2025) reported a summary correlation of *r* = −0.204 in a global MASEM study, both closely matching our result and supporting the cross-cultural generalizability of this association. The slight difference from Zeng et al. (2022) may be attributable to our broader sample, which included junior and senior high school students in addition to college students. Individual large-sample studies, like those by Yang et al. (2020) (*r* = −0.21) and Tong et al. (2022) (*r* = −0.18), fall within our 95% CI, indicating our pooled effect accurately reflects the situation among Chinese adolescents.

Although the effect magnitude is small-to-medium, its practical significance is substantial given the high prevalence of mobile phone addiction (27.8%) and physical inactivity (>60% insufficiently active) among Chinese adolescents (Wan and Ren, 2023). Even small increases in physical activity at the population level could help a large number of adolescents. The mechanisms likely include: (1) Time substitution: physical activity occupies free time, reducing time available for phone use and breaking the sedentary-phone use cycle. (2) Neurotransmitter effects: sustained exercise releases dopamine, serotonin, and endorphins, enhancing brain reward system function and reducing craving for virtual rewards from phones. (3) Mood improvement: exercise alleviates anxiety and depression, reducing the tendency to use phones for escapism (Kim and Ahn, 2023).

#### 4.1.2 Association between physical activity and psychological resilience

This study found a significant positive correlation between adolescent physical activity and psychological resilience (*r* = 0.370, 95% CI: 0.250 to 0.479), a medium effect size. This aligns with a 2025 MASEM study by Cui et al., 2025 (the summary correlation of *r* = 0.26), demonstrating high cross-study consistency. Single-sample domestic studies also report similar correlations (e.g., Shen and Gao, 2024; *r* = 0.37). The mechanisms linking physical activity to resilience are multi-faceted. Physiologically, moderate activity promotes neurogenesis in the hippocampus and is associated with higher prefrontal cortex executive function, enhancing emotional regulation and stress coping. Psychologically, achieving goals in activity increases self-efficacy and confidence (Kim and Ahn, 2023). Team sports foster social skills and resilience to failure, while overcoming challenges during exercise cultivates persistence and positive attitudes.

#### 4.1.3 The heterogeneity for effect size

Heterogeneity was extremely high in all three pairwise meta-analyses (PA-MPA: I^2^ = 98.46%, k = 42; PA-PR: I^2^ = 98.40%, k = 13; PR-MPA: I^2^ = 99.44%, k = 8), indicating that true effects vary substantially across studies and that the pooled values should be read as overall tendencies rather than precise point estimates. Importantly, the direction of every association was consistent across this variability - physical activity was negatively associated with mobile phone addiction and positively with psychological resilience, and resilience was negatively associated with addiction - suggesting that the associations are robust in direction even if their magnitude is context-dependent. Subgroup analyses and meta-regressions located several sources of this variability. Education level significantly moderated only the PR–MPA relationship (Qb = 5.56, *p* = 0.018), study design moderated the PA–MPA link (cross-sectional *r* = −0.237 vs. longitudinal *r* = −0.120, Qb = 8.23, *p* = 0.004), and the physical-activity instrument moderated the PA–PR relationship (Qb = 6.21, *p* = 0.013); in contrast, region, education level (for PA–MPA and PA–PR), and the PA instrument (for PA–MPA) did not significantly account for heterogeneity. The percentage of male participants explained 10.3% of the variance, whereas mean age and publication year did not (R^2^ ~ 0%, all *p* > 0.20). Because a substantial share of heterogeneity remained unexplained, future primary studies should report sample characteristics—particularly gender composition and publication type—in greater detail to permit finer moderator analyses.

#### 4.1.4 The mediating role of psychological resilience

Using MASEM, this study quantitatively tested the mediating role of psychological resilience for the first time at the meta-analytic level. The results confirmed a significant mediating effect (indirect effect = −0.120, accounting for 53.3%of total effect). This aligns generally with domestic single-sample studies, though the effect size differs somewhat. Previous studies reported smaller proportions, such as Shen and Gao (2024) (18.3%) and Zhao et al. (2022) (15.2%). Compared to another key mediator, Lin et al. (2025) MASEM study found self-control accounted for 49.7% of the effect, suggesting self-control might be a more central psychological mechanism in the physical activity-phone addiction link. Discrepancies in mediation proportions may arise from: (1) Methodology: Meta-analysis synthesizes heterogeneous studies, while single-sample studies have higher internal consistency. (2) Measurement tools: Different resilience scales (e.g., CD-RISC vs. RS-14) have varying factor structures and psychometric properties. (3) Model choice: Some studies use chain mediation models, while this study examined only the single mediation of resilience.

The mediation proportion (53.3%) raises the important question of what additional mechanisms may account for the remaining relationship between physical activity and mobile phone addiction. Several alternative pathways have been identified in the literature: (1) Self-control: Lin et al. (2025) found that self-control accounted for 49.7% of the PA–MPA relationship in a MASEM study, suggesting it may be a more prominent mediator. (2) Sleep quality: Kumar et al. (2024) reported Physical activity, sleep quality, and mobile phone addiction are empirically interrelated—poor sleep co-occurs with both low physical activity and high phone addiction. (3) Mood regulation: Physical activity is associated with lower anxiety and depression, which are known risk factors for mobile phone addiction. (4) Time displacement: The simplest mechanism—time spent in physical activity is time not spent on phones. These mechanisms likely operate in parallel and may interact with each other. Future research should employ multiple-mediator models to simultaneously test these pathways.

### 4.2 Practical implications

The findings of this study have practical implications for adolescent health promotion, although they should be considered in light of the predominantly observational evidence base. At the school level, integrating physical activity promotion with psychological resilience training may be a promising approach, but its effectiveness should be tested in future randomized controlled trials. At the family level, parents can model healthy behaviors by limiting their own phone use and engaging in family physical activities. At the individual level, adolescents should be encouraged to maintain regular physical activity and develop resilience skills. It is important to emphasize that these recommendations are based on observational evidence, and the effectiveness of combined “physical activity plus psychological intervention” programs should be empirically tested before being widely implemented.

### 4.3 Study limitations

This study has limitations. First, the included studies are predominantly cross-sectional (54 out of 58). Causal relationships cannot be firmly established; only associations are demonstrated. Second, the generalisability of the findings is limited by the composition of the evidence base. Forty-nine of the 58 included studies (84.5%) were conducted in China (including Taiwan and Hong Kong), and 42 (72.4%) sampled university students, with comparatively few studies from other cultural regions, primary/secondary school populations, or non-student adolescents. Consequently, the pooled estimates should be interpreted as most applicable to Chinese university and secondary-school adolescents, and caution is warranted when extrapolating to other cultural contexts, younger age groups, or adult populations. Greater geographic and developmental diversity in future primary research is needed before the mediation model can be considered universally robust. Third, the study only examined the single mediator of psychological resilience, ignoring other potential mediators or moderators, thus providing an incomplete picture of the mechanisms linking physical activity to phone addiction.

## 5 Conclusion

Using traditional meta-analysis and MASEM, this study synthesized 58 studies involving 98,946 adolescents and young adults to examine the relationships among physical activity (PA), psychological resilience (PR), and mobile phone addiction (MPA). The main findings are as follows: PA is negatively correlated with MPA (the summary correlation of *r* = −0.225), supporting PA as a small-to-medium factor associated with lower MPA, possibly via time displacement, neurotransmitter regulation, and mood improvement. PA is positively correlated with PR (the summary correlation of *r* = 0.370, medium effect), indicating regular exercise is associated with higher resilience development. PR mediates the PA–MPA relationship (indirect effect = −0.120, accounting for 53.3% of the total effect). Thus, PA is indirectly associated with lower MPA through its positive relationship with resilience, though other mechanisms likely exist. This is the first MASEM study to quantify the mediating role of psychological resilience. The findings suggest that combined PA-plus-psychological approaches merit testing in longitudinal and experimental studies. Efforts should simultaneously promote physical activity and foster psychological resilience.

## Data Availability

The original contributions presented in the study are included in the article/Supplementary material, further inquiries can be directed to the corresponding author.
