# Supplementary material for: Physical activity, psychological resilience, and adolescent mobile phone addiction—a meta-analytic structural equation modeling study
Source: Front Psychol. 2026 Oct 2;17:1914677. doi: 10.3389/fpsyg.2026.1914677 (PMC13672197; doi:10.3389/fpsyg.2026.1914677)
Supplement: Supplementary file 1 [file Supplementary_file_1.docx]

Supplementary Material

[**Search strategy**	1](#_Toc231330533)

[**Summary of characteristics of studies included in the meta-analysis**	6](#_Toc231330534)

[**Research quality assessment**	17](#_Toc231330535)

[**Forest plot**	22](#_Toc231330536)

[**Publication bias**	22](#_Toc231330537)

**Search strategy**

**Table S1. Web of Science Search Results**

|  | Search Query | Results |
| --- | --- | --- |
| 1 | (((AB=(physical activit*)) OR AB=(exercis*)) OR AB=(sport*)) OR AB=(training) Editions: WOS.SCI,WOS.SSCI | 124589 |
| 2 | ((((AB=(mobile phone addict*)) OR AB=(smartphone addict*)) OR AB=(phone addiction)) OR AB=(mobile phone overuse)) Editions: WOS.SCI,WOS.SSCI | 1896 |
| 3 | (((AB=(psychological resilience)) OR AB=(resilience)) OR AB=(resilient)) | 2168 |
| 4 | #3 AND #2, or #3 AND #1, or #1 AND #2 | 268 |

May 5, 2026

**Table. S2 PsycINFO Search Results**

|  | Search Query | Results |
| --- | --- | --- |
| 1 | #1 AB (exercis# OR "physical activit#" OR sport# OR training) AND AB ("mobile phone addict#" OR "smartphone addict#" OR "phone addiction" OR "mobile phone overuse")  #2 AB (exercis# OR "physical activit#" OR sport# OR training) AND AB ("psychological resilience" OR resilience OR resilient)  #3 AB ("mobile phone addict#" OR "smartphone addict#" OR "phone addiction" OR "mobile phone overuse") AND AB ("psychological resilience" OR resilience OR resilient) | 284 |

May 5, 2026

**Table S3. SPORTDiscus Search Results**

|  | Search Query | Results |
| --- | --- | --- |
| 1 | #1 AB (exercis# OR "physical activit#" OR sport# OR training) AND AB ("mobile phone addict#" OR "smartphone addict#" OR "phone addiction" OR "mobile phone overuse")  #2 AB (exercis# OR "physical activit#" OR sport# OR training) AND AB ("psychological resilience" OR resilience OR resilient)  #3 AB ("mobile phone addict#" OR "smartphone addict#" OR "phone addiction" OR "mobile phone overuse") AND AB ("psychological resilience" OR resilience OR resilient) | 293 |

May 5, 2026

**Table S4. PubMed Search Results**

|  | Search Details | Results |
| --- | --- | --- |
| 1 | "exercis*"[Title/Abstract] OR "physical activit*"[Title/Abstract] OR "sport*"[Title/Abstract] OR "training"[Title/Abstract] | 1,167,385 |
| 2 | "mobile phone addict*"[Title/Abstract] OR "smartphone addict*"[Title/Abstract] OR "phone addiction"[Title/Abstract] OR "mobile phone overuse"[Title/Abstract] | 7896 |
| 3 | "psychological resilience"[Title/Abstract] OR resilience[Title/Abstract] OR resilient[Title/Abstract] | 92658 |
| 4 | #3 AND #2, or #3 AND #1, or #1 AND #2, | 420 |

May 5, 2026

**Table S5. CNKI Search Results**

|  | Search Details | Results |
| --- | --- | --- |
| 1 | (身体活动 + 运动 + 锻炼) AND (手机成瘾 + 智能手机成瘾 + 手机过度使用)  or (心理韧性 + 抗逆力) AND (身体活动 + 运动 + 锻炼)  or (心理韧性 + 抗逆力) AND (手机成瘾 + 智能手机成瘾 + 手机过度使用) | 103 |

May 5, 2026

**Table S6： Summary of characteristics of studies included in the meta-analysis**

**Supplement S6 - Characteristics of Included Studies (63 records across the three relationship pairs)**

*The effect size and its 95% confidence interval are reported in a single cell.*

| Relationship pair | Study | Year | Country/Region | Design | Education level | N | Mean age | % male | PA measure | Outcome measure | r [95% CI] | Fisher's z | SE |
| --- | --- | --- | --- | --- | --- | --- | --- | --- | --- | --- | --- | --- | --- |
| PA-MPA | Cao et al. (2023) | 2023 | China | Cross-sectional | Junior high | 445 | 13.00 | 46.1 | PARS-3 | SAS | -0.169 [-0.258, -0.077] | -0.1706 | 0.0476 |
| PA-MPA | Ceylan & Demirdel (2023) | 2023 | Turkiye | Cross-sectional | University | 424 | 20.30 | 23.1 | IPAQ | SAS-SV | -0.045 [-0.140, 0.050] | -0.0450 | 0.0487 |
| PA-MPA | Cetin et al. (2023) | 2023 | Turkiye | Cross-sectional | Young adults | 86 | 22.54 | 46.5 | IPAQ | SAS | -0.129 [-0.332, 0.085] | -0.1297 | 0.1098 |
| PA-MPA | Chao et al. (2022) | 2022 | Taiwan, China | Cross-sectional | University | 1575 | 19.50 | 48.5 | Self-report frequency | SAS | -0.040 [-0.089, 0.009] | -0.0400 | 0.0252 |
| PA-MPA | Chen & Zhang (2021) | 2021 | China | Cross-sectional | University | 1898 |  | 45.5 | IPAQ | MPATS | -0.460 [-0.495, -0.424] | -0.4973 | 0.0230 |
| PA-MPA | Chen H. et al. (2022) | 2022 | China | Cross-sectional | University | 9406 | 19.60 |  | IPAQ | MPAI | -0.060 [-0.080, -0.040] | -0.0601 | 0.0103 |
| PA-MPA | Ding et al. (2021) | 2021 | China | Cross-sectional | University | 1786 | 19.00 |  | PARS-3 | MPATS | -0.445 [-0.481, -0.407] | -0.4784 | 0.0237 |
| PA-MPA | Dong et al. (2023) | 2023 | China | Cross-lagged | Junior high | 882 |  |  | PARS-3 | MPAI | -0.091 [-0.156, -0.025] | -0.0913 | 0.0337 |
| PA-MPA | Gao et al. (2023) | 2023 | China | Cross-sectional | University | 1019 | 19.50 |  | PARS-3 | MPATS | -0.255 [-0.312, -0.197] | -0.2608 | 0.0314 |
| PA-MPA | Gong et al. (2023) | 2023 | China | Cross-sectional | University | 643 | 19.68 | 56.5 | PARS-3 | SAS | -0.300 [-0.369, -0.228] | -0.3095 | 0.0395 |
| PA-MPA | Guo et al. (2022) | 2022 | China | Cross-sectional | University | 1433 | 19.67 | 49.1 | PARS-3 | MPATS | -0.158 [-0.208, -0.107] | -0.1593 | 0.0264 |
| PA-MPA | Han et al. (2023) | 2023 | China | Cross-sectional | University | 4959 |  | 54.4 | PARS-3 | MPATS | -0.279 [-0.304, -0.253] | -0.2866 | 0.0142 |
| PA-MPA | HariPriya et al. (2019) | 2019 | India | Cross-sectional | University | 113 | 21.00 | 44.2 | IPAQ | SAS | -0.335 [-0.489, -0.160] | -0.3484 | 0.0953 |
| PA-MPA | Huang et al. (2022) | 2022 | Taiwan, China | Longitudinal | University | 452 | 23.70 | 40.7 | IPAQ | SABAS | -0.021 [-0.113, 0.071] | -0.0210 | 0.0472 |
| PA-MPA | Kim & Ahn (2023) | 2023 | Korea | Longitudinal | Junior/Senior high | 2242 | 14.00 |  | Self-report | SAPS | -0.176 [-0.216, -0.136] | -0.1779 | 0.0211 |
| PA-MPA | Kim S.-E. et al. (2015) | 2015 | Korea | Cross-sectional | University | 110 | 21.03 | 60.9 | Accelerometer | SAPS | -0.578 [-0.691, -0.438] | -0.6595 | 0.0967 |
| PA-MPA | Kumar et al. (2024) | 2024 | India | Cross-sectional | University | 138 | 21.00 |  | IPAQ | SAS | -0.377 [-0.512, -0.224] | -0.3966 | 0.0861 |
| PA-MPA | Jin C. et al. (2024) | 2024 | China | Cross-sectional | University | 930 |  | 29.2 | PARS-3 | MPDI | -0.133 [-0.196, -0.069] | -0.1338 | 0.0328 |
| PA-MPA | Li X.-P. et al. (2023) | 2023 | China | Cross-sectional | University | 509 | 18.81 | 37.9 | PARS-3 | MPATS | -0.220 [-0.301, -0.136] | -0.2237 | 0.0445 |
| PA-MPA | Lin B. et al. (2022) | 2022 | China | Cross-sectional | University | 1787 | 18.85 | 35.1 | GLTEQ | SAS-SV | -0.153 [-0.198, -0.107] | -0.1542 | 0.0237 |
| PA-MPA | Liu & Sun (2023) | 2023 | China | Cross-sectional | University | 488 | 19.21 | 75.8 | PARS-3 | MPATS | -0.210 [-0.293, -0.124] | -0.2132 | 0.0454 |
| PA-MPA | Lu T. et al. (2022) | 2022 | China | Cross-sectional | University | 9569 | 19.50 | 50.2 | IPAQ | MPAI | -0.058 [-0.078, -0.038] | -0.0581 | 0.0102 |
| PA-MPA | Niu S. (2023) | 2023 | China | Cross-sectional | University | 514 | 18.43 | 43.2 | PARS-3 | MPAS | -0.100 [-0.185, -0.014] | -0.1003 | 0.0442 |
| PA-MPA | Numanoglu-Akbas et al. (2020) | 2020 | Turkiye | Cross-sectional | University | 288 | 19.97 | 44.8 | IPAQ | SAS-SV | -0.126 [-0.238, -0.011] | -0.1267 | 0.0592 |
| PA-MPA | Sezer Efe et al. (2023) | 2023 | Turkiye | Cross-sectional | Senior high | 437 | 16.30 | 20.1 | IPAQ | SAS-SV | -0.107 [-0.199, -0.013] | -0.1074 | 0.0480 |
| PA-MPA | Shen B. (2024) | 2024 | China | Cross-sectional | University | 802 |  | 41.4 | PARS-3 | MPATS | -0.233 [-0.297, -0.166] | -0.2374 | 0.0354 |
| PA-MPA | Su et al. (2024) | 2024 | China | Cross-sectional | University | 913 | 21.10 | 47.4 | Self-report frequency | SABAS | -0.165 [-0.227, -0.101] | -0.1665 | 0.0331 |
| PA-MPA | Zhang D. et al. (2022) | 2022 | China | Cross-sectional | Mixed (primary-university) | 649 |  | 48.7 | PARS-3 | CPDS | -0.223 [-0.295, -0.149] | -0.2268 | 0.0393 |
| PA-MPA | Tong & Meng (2023) | 2023 | China | Cross-sectional | University | 4399 | 19.20 | 56.4 | PARS-3 | MPAI | -0.713 [-0.727, -0.698] | -0.8933 | 0.0151 |
| PA-MPA | Tong W.-X. et al. (2022) | 2022 | China | Cross-sectional | University | 3609 |  |  | IPAQ | MPATS | -0.173 [-0.204, -0.141] | -0.1748 | 0.0167 |
| PA-MPA | Wan & Ren (2023) | 2023 | China | Cross-sectional | Mixed (primary-junior) | 516 | 12.13 | 48.1 | PARS-3 | MPAI | -0.278 [-0.356, -0.196] | -0.2855 | 0.0442 |
| PA-MPA | Wang J. & Hao (2023) | 2023 | China | Cross-sectional | University | 1112 | 21.40 | 38.9 | Self-report frequency | MPAI | -0.099 [-0.157, -0.040] | -0.0993 | 0.0300 |
| PA-MPA | Wang J. et al. (2024) | 2024 | China | Cross-sectional | University | 301 | 20.47 | 57.8 | PARS-3 | MPATS | -0.120 [-0.230, -0.007] | -0.1206 | 0.0579 |
| PA-MPA | Wei Z. (2023) | 2023 | China | Cross-sectional | University | 1013 |  | 69.5 | PARS-3 | MPATS | -0.270 [-0.326, -0.212] | -0.2769 | 0.0315 |
| PA-MPA | Xiao S. (2022) | 2022 | China | Cross-sectional | University | 3122 | 18.91 | 19.5 | Self-report | MPATS | -0.113 [-0.147, -0.078] | -0.1135 | 0.0179 |
| PA-MPA | Xu C.-Y. et al. (2023) | 2023 | China | Cross-sectional | University | 5075 | 20.00 |  | PARS-3 | MPATS | -0.165 [-0.192, -0.138] | -0.1665 | 0.0140 |
| PA-MPA | Yang G. et al. (2019) | 2019 | China | Cross-sectional | University | 608 | 20.06 | 26.0 | PARS-3 | MPATS | -0.124 [-0.202, -0.045] | -0.1246 | 0.0407 |
| PA-MPA | Zeng M. et al. (2022) | 2022 | China | Cross-sectional | University | 1843 | 19.75 | 42.8 | PARS-3 | MPATS | -0.140 [-0.184, -0.095] | -0.1409 | 0.0233 |
| PA-MPA | Zhang L.-Q. & Gao H.-N. (2023) | 2023 | China | Cross-sectional | Senior high | 560 | 16.56 | 53.6 | PARS-3 | MPAI | -0.747 [-0.781, -0.708] | -0.9661 | 0.0424 |
| PA-MPA | Zhao X. et al. (2024) | 2024 | China | Longitudinal | Junior/Senior high | 2131 | 12.14 | 48.5 | Self-report frequency | SAPS | -0.140 [-0.181, -0.098] | -0.1409 | 0.0217 |
| PA-MPA | Zheng & Ma (2020) | 2020 | China | Cross-sectional | University | 418 |  |  | PARS-3 | MPATS | -0.134 [-0.227, -0.039] | -0.1348 | 0.0491 |
| PA-MPA | Zhu L. et al. (2023) | 2023 | China | Cross-sectional | University | 823 | 18.55 | 41.4 | IPAQ | SAS-SV | -0.151 [-0.217, -0.084] | -0.1522 | 0.0349 |
| PA-PR | Belaire et al. (2024) | 2024 | USA | Cross-sectional | Primary school | 523 | 10.00 |  | PAQ-C | CD-RISC | +0.294 [0.214, 0.370] | 0.3029 | 0.0439 |
| PA-PR | Cui W. (2022) | 2022 | China | Cross-sectional | University | 824 |  |  | PARS-3 | CD-RISC | +0.420 [0.362, 0.475] | 0.4477 | 0.0349 |
| PA-PR | Ho et al. (2015) | 2015 | Hong Kong, China | Cross-sectional | Junior/Senior high | 775 | 12.28 |  | PARCY | CD-RISC | +0.250 [0.183, 0.315] | 0.2554 | 0.0360 |
| PA-PR | Li H.-Y. et al. (2022) | 2022 | China | Cross-sectional | Primary school | 1009 | 11.00 | 51.4 | Self-report | RSCA | +0.234 [0.175, 0.292] | 0.2384 | 0.0315 |
| PA-PR | Li et al. (2021) | 2021 | China | Cross-sectional | University | 1214 |  |  | PARS-3 | CD-RISC | +0.470 [0.425, 0.513] | 0.5101 | 0.0287 |
| PA-PR | Liu C.-H. (2020) | 2020 | China | Cross-sectional | University | 1408 | 20.12 | 51.7 | PARS-3 | RSCA | +0.359 [0.313, 0.404] | 0.3757 | 0.0267 |
| PA-PR | Meng Q.-R. et al. (2024) | 2024 | China | Cross-sectional | University | 746 |  |  | PARS-3 | CD-RISC | +0.656 [0.613, 0.695] | 0.7858 | 0.0367 |
| PA-PR | Shen B. (2024) | 2024 | China | Cross-sectional | University | 802 |  | 41.4 | PARS-3 | CD-RISC | +0.368 [0.307, 0.426] | 0.3861 | 0.0354 |
| PA-PR | Zhang Z. et al. (2022) | 2022 | China | Cross-sectional | University | 1117 | 18.90 | 49.6 | IPAQ | CD-RISC | +0.098 [0.040, 0.156] | 0.0983 | 0.0300 |
| PA-PR | Zhang M et al. (2023) | 2023 | China | Cross-sectional | Junior high | 3143 | 12.88 | 47.8 | Self-report | CD-RISC | +0.249 [0.216, 0.282] | 0.2543 | 0.0178 |
| PA-PR | Zhang L.-Q. & Gao H.-N. (2023) | 2023 | China | Cross-sectional | Senior high | 560 | 16.56 | 53.6 | PARS-3 | RSCA | +0.765 [0.728, 0.797] | 1.0082 | 0.0424 |
| PA-PR | Zhao Z. et al. (2022) | 2022 | China | Cross-sectional | University | 257 |  | 28.4 | PARS-3 | CD-RISC | +0.195 [0.074, 0.310] | 0.1975 | 0.0627 |
| PA-PR | Zhou H. (2022) | 2022 | China | Cross-sectional | University | 722 |  | 53.6 | PARS-3 | BRS | +0.190 [0.119, 0.259] | 0.1923 | 0.0373 |
| MPA-PR | Hu B. et al. (2024) | 2024 | China | Cross-sectional | University | 5048 |  | 50.3 | MPAI | CD-RISC | -0.099 [-0.126, -0.072] | -0.0993 | 0.0141 |
| MPA-PR | Xie G. et al. (2023) | 2023 | China | Cross-sectional | University | 7234 | 18.86 | 29.8 | MPAI | CD-RISC | -0.132 [-0.155, -0.109] | -0.1328 | 0.0118 |
| MPA-PR | Ma A. et al. (2022) | 2022 | China | Cross-sectional | Junior/Senior high | 2268 | 14.90 | 43.2 | MPAI | RSCA | -0.405 [-0.439, -0.370] | -0.4296 | 0.0210 |
| MPA-PR | Shen B. (2024) | 2024 | China | Cross-sectional | University | 802 |  | 41.4 | MPATS | CD-RISC | -0.320 [-0.381, -0.256] | -0.3316 | 0.0354 |
| MPA-PR | Tian F. et al. (2025) | 2025 | China | Cross-sectional | Junior/Senior high | 401 | 14.56 | 77.0 | MPATS | CD-RISC-10 | -0.327 [-0.412, -0.237] | -0.3395 | 0.0501 |
| MPA-PR | Wu J.-X. et al. (2024) | 2024 | China | Cross-sectional | Junior/Senior high | 2230 | 14.92 | 43.1 | MPAI | RSCA | -0.376 [-0.411, -0.340] | -0.3954 | 0.0212 |
| MPA-PR | Zhao Z. et al. (2022) | 2022 | China | Cross-sectional | University | 257 |  | 28.4 | MPAI | CD-RISC | -0.250 [-0.361, -0.132] | -0.2554 | 0.0627 |
| MPA-PR | Zhang L.-Q. & Gao H.-N. (2023) | 2023 | China | Cross-sectional | Senior high | 560 | 16.56 | 53.6 | MPAI | RSCA | -0.786 [-0.816, -0.752] | -1.0609 | 0.0424 |

**Table S7 Quality Assessment of Included Studies (58 studies)**

| Study (Year) | Design | 1. | 2. | 3. | 4. | 5. | 6. | 7. | 8. | 9. | Total/9 | Grade |
| --- | --- | --- | --- | --- | --- | --- | --- | --- | --- | --- | --- | --- |
| Hu B. et al. (2024) | Cross-sect | No | Yes | No | No | Yes | Yes | Yes | No | Yes | 5 | Moderate |
| Ma A. et al. (2022) | Cross-sect | No | Yes | No | No | Yes | Yes | Yes | No | Yes | 5 | Moderate |
| Shen B. (2024) | Cross-sect | No | Yes | No | Yes | Yes | Yes | Yes | No | Yes | 6 | Moderate |
| Tian F. et al. (2025) | Cross-sect | No | No | No | Yes | Yes | Yes | Yes | Yes | Yes | 6 | Moderate |
| Wu J.-X. et al. (2024) | Cross-sect | Yes | Yes | No | Yes | Yes | Yes | Yes | No | Yes | 7 | High |
| Xie G. et al. (2023) | Cross-sect | No | Yes | No | No | Yes | Yes | Yes | No | Yes | 5 | Moderate |
| Zhang L.-Q. & Gao H.-N. (2023) | Cross-sect | Yes | Yes | No | No | Yes | Yes | Yes | No | Yes | 6 | Moderate |
| Zhao Z. et al. (2022) | Cross-sect | No | No | No | No | Yes | Yes | Yes | No | No | 3 | Low |
| Cao et al. (2023) | Cross-sect | No | No | Yes | No | Yes | Yes | Yes | No | Yes | 5 | Moderate |
| Cetin et al. (2023) | Cross-sect | Yes | No | Yes | No | Yes | Yes | Yes | Yes | Yes | 7 | High |
| Ceylan & Demirdel (2023) | Cross-sect | No | No | Yes | No | Yes | Yes | Yes | No | Yes | 5 | Moderate |
| Chao et al. (2022) | Cross-sect | No | Yes | No | Yes | Yes | Yes | Yes | Yes | Yes | 7 | High |
| Chen & Zhang (2021) | Cross-sect | Yes | Yes | Yes | Yes | Yes | Yes | Yes | Yes | Yes | 9 | High |
| Chen H. et al. (2022) | Cross-sect | No | Yes | Yes | Yes | Yes | Yes | Yes | No | Yes | 7 | High |
| Ding et al. (2021) | Cross-sect | No | Yes | Yes | No | Yes | Yes | Yes | Yes | No | 6 | Moderate |
| Dong et al. (2023) | Cross-lagg | Yes | Yes | Yes | Yes | Yes | Yes | Yes | Yes | Yes | 9 | High |
| Gao et al. (2023) | Cross-sect | No | Yes | Yes | Yes | Yes | Yes | Yes | No | Yes | 7 | High |
| Gong et al. (2023) | Cross-sect | No | Yes | Yes | No | Yes | Yes | Yes | No | Yes | 6 | Moderate |
| Guo et al. (2022) | Cross-sect | Yes | Yes | Yes | Yes | Yes | Yes | Yes | No | Yes | 8 | High |
| Han et al. (2023) | Cross-sect | Yes | Yes | Yes | No | Yes | Yes | Yes | No | Yes | 7 | High |
| HariPriya et al. (2019) | Cross-sect | No | No | Yes | Yes | Yes | Yes | Yes | Yes | Yes | 7 | High |
| Huang et al. (2022) | Longitudin | Yes | No | Yes | Yes | Yes | Yes | Yes | Yes | Yes | 8 | High |
| Jin C. et al. (2024) | Cross-sect | Yes | Yes | Yes | No | Yes | Yes | Yes | No | Yes | 7 | High |
| Kim & Ahn (2023) | Longitudin | No | Yes | No | No | Yes | Yes | Yes | Yes | Yes | 6 | Moderate |
| Kim S.-E. et al. (2015) | Cross-sect | Yes | No | Yes | No | Yes | Yes | Yes | No | Yes | 6 | Moderate |
| Kumar et al. (2024) | Cross-sect | Yes | No | Yes | No | Yes | Yes | Yes | No | Yes | 6 | Moderate |
| Li X.-P. et al. (2023) | Cross-sect | No | Yes | Yes | Yes | Yes | Yes | Yes | Yes | Yes | 8 | High |
| Lin B. et al. (2022) | Cross-sect | No | Yes | Yes | Yes | Yes | Yes | Yes | Yes | No | 7 | High |
| Liu & Sun (2023) | Cross-sect | No | No | Yes | Yes | Yes | Yes | Yes | No | Yes | 6 | Moderate |
| Lu T. et al. (2022) | Cross-sect | Yes | Yes | Yes | No | Yes | Yes | Yes | No | No | 6 | Moderate |
| Niu S. (2023) | Cross-sect | No | Yes | Yes | Yes | Yes | Yes | Yes | Yes | Yes | 8 | High |
| Numanoglu-Akbas et al. (2020) | Cross-sect | No | No | Yes | No | Yes | Yes | Yes | No | Yes | 5 | Moderate |
| Sezer Efe et al. (2023) | Cross-sect | Yes | No | Yes | Yes | Yes | Yes | Yes | Yes | Yes | 8 | High |
| Su et al. (2024) | Cross-sect | Yes | Yes | No | No | Yes | Yes | Yes | No | No | 5 | Moderate |
| Tong & Meng (2023) | Cross-sect | Yes | Yes | Yes | No | Yes | Yes | Yes | No | Yes | 7 | High |
| Tong W.-X. et al. (2022) | Cross-sect | No | Yes | Yes | No | Yes | Yes | Yes | No | Yes | 6 | Moderate |
| Wan & Ren (2023) | Cross-sect | No | Yes | Yes | No | Yes | Yes | Yes | No | Yes | 6 | Moderate |
| Wang J. & Hao (2023) | Cross-sect | No | Yes | No | No | Yes | Yes | Yes | No | Yes | 5 | Moderate |
| Wang J. et al. (2024) | Cross-sect | Yes | No | Yes | No | Yes | Yes | Yes | Yes | Yes | 7 | High |
| Wei Z. (2023) | Cross-sect | No | Yes | Yes | Yes | Yes | Yes | Yes | No | No | 6 | Moderate |
| Xiao S. (2022) | Cross-sect | No | Yes | No | Yes | Yes | Yes | Yes | Yes | No | 6 | Moderate |
| Xu C.-Y. et al. (2023) | Cross-sect | Yes | Yes | Yes | No | Yes | Yes | Yes | No | Yes | 7 | High |
| Yang G. et al. (2019) | Cross-sect | No | Yes | Yes | No | Yes | Yes | Yes | No | Yes | 6 | Moderate |
| Zeng M. et al. (2022) | Cross-sect | No | Yes | Yes | Yes | Yes | Yes | Yes | Yes | No | 7 | High |
| Zhang D. et al. (2022) | Cross-sect | No | Yes | Yes | Yes | Yes | Yes | Yes | Yes | Yes | 8 | High |
| Zhao X. et al. (2024) | Longitudin | Yes | Yes | No | No | Yes | Yes | Yes | Yes | Yes | 7 | High |
| Zheng & Ma (2020) | Cross-sect | No | No | Yes | No | Yes | Yes | Yes | No | No | 4 | Moderate |
| Zhu L. et al. (2023) | Cross-sect | Yes | Yes | Yes | No | Yes | Yes | Yes | Yes | Yes | 8 | High |
| Belaire et al. (2024) | Cross-sect | No | Yes | Yes | No | Yes | Yes | Yes | Yes | Yes | 7 | High |
| Cui W. (2022) | Cross-sect | Yes | Yes | Yes | No | Yes | Yes | Yes | No | No | 6 | Moderate |
| Ho et al. (2015) | Cross-sect | No | Yes | No | No | Yes | Yes | Yes | No | Yes | 5 | Moderate |
| Li H.-Y. et al. (2022) | Cross-sect | No | Yes | No | Yes | Yes | Yes | Yes | No | Yes | 6 | Moderate |
| Li et al. (2021) | Cross-sect | Yes | Yes | Yes | Yes | Yes | Yes | Yes | Yes | Yes | 9 | High |
| Liu C.-H. (2020) | Cross-sect | Yes | Yes | Yes | No | Yes | Yes | Yes | No | Yes | 7 | High |
| Meng Q.-R. et al. (2024) | Cross-sect | Yes | Yes | Yes | Yes | Yes | Yes | Yes | No | Yes | 8 | High |
| Zhang Z. et al. (2022) | Cross-sect | No | Yes | Yes | No | Yes | Yes | Yes | Yes | Yes | 7 | High |
| Zhang et al. (2023) | Cross-sect | Yes | Yes | No | No | Yes | Yes | Yes | Yes | Yes | 7 | High |
| Zhou H. (2022) | Cross-sect | No | Yes | Yes | Yes | Yes | Yes | Yes | No | No | 6 | Moderate |

Note: Quality was assessed with the Newcastle-Ottawa Scale (NOS; Wells et al., 2009), framed as Selection (items 1–4), Comparability (items 5–6) and Outcome (items 7–9), maximum 9 points. Grades: High = 7–9, Moderate = 4–6, Low = 0–3. Because 54 of the 58 studies were cross-sectional, the cohort-oriented items were translated into the following design-equivalent criteria: Item 1 (Representativeness) – the sampling strategy was random, stratified, or cluster-based and did not rely solely on a convenience sample; Item 2 (Selection) – sample size ≥ 500; Item 3 (Ascertainment of exposure) – physical activity was assessed with a validated instrument (e.g., PARS-3, IPAQ); Item 4 (Outcome not present at baseline) – the response or valid-questionnaire rate was reported; Items 5–6 (Comparability) – the study statistically adjusted for at least one relevant covariate (e.g., age, sex, body-mass index) or otherwise ensured comparability; Item 7 (Outcome assessment) – mobile phone addiction was measured with a validated instrument; Item 8 (Follow-up duration) – the temporal structure of data collection (e.g., single-wave or multi-wave measurement) was explicitly documented; Item 9 (Follow-up completeness) – an effect size with its 95% confidence interval, or sufficient statistics for its computation, was reported. An item was credited only when the criterion was explicitly reported in the primary study and never inferred from indirect information. This conservative rule explains the distribution of deductions: representativeness, response-rate reporting, and documentation of the temporal structure were the least frequently met criteria (41.4%, 41.4%, and 39.7%, respectively), whereas the comparability and outcome-assessment items were met by all studies, because statistical adjustment for covariates and the use of validated instruments were universal practices in this literature.

**Table S8. MASEM analysis details**

This supplementary table provides the study-level data and the full reproducibility materials for the two-stage meta-analytic structural equation modeling (MASEM) reported in the manuscript. Part A lists, for each of the 58 included studies, the reported pairwise correlation(s), sample size(s), and a unique analysis identifier. A Note explains how missing correlations were handled. Part B reports the Stage-1 pooled correlation vector and its asymptotic covariance matrix. Part C gives the Stage-2 model specification. Part D gives convergence information, and Part E describes the bootstrap procedure.

**Part A. Study-level correlation matrices and sample sizes**

| **ID** | **Study (Author, Year)** | **PA–MPA r [95% CI]** | **PA–PR r [95% CI]** | **PR–MPA r [95% CI]** | **N** | **# pairs** |
| --- | --- | --- | --- | --- | --- | --- |
| S1 | Cao et al. (2023) | -0.169 [-0.258, -0.077] |  |  | 445 | 1 |
| S2 | Ceylan & Demirdel (2023) | -0.045 [-0.140, 0.050] |  |  | 424 | 1 |
| S3 | Cetin et al. (2023) | -0.129 [-0.332, 0.085] |  |  | 86 | 1 |
| S4 | Chao et al. (2022) | -0.040 [-0.089, 0.009] |  |  | 1575 | 1 |
| S5 | Chen & Zhang (2021) | -0.460 [-0.495, -0.424] |  |  | 1898 | 1 |
| S6 | Chen H. et al. (2022) | -0.060 [-0.080, -0.040] |  |  | 9406 | 1 |
| S7 | Ding et al. (2021) | -0.445 [-0.481, -0.407] |  |  | 1786 | 1 |
| S8 | Dong et al. (2023) | -0.091 [-0.156, -0.025] |  |  | 882 | 1 |
| S9 | Gao et al. (2023) | -0.255 [-0.312, -0.197] |  |  | 1019 | 1 |
| S10 | Gong et al. (2023) | -0.300 [-0.369, -0.228] |  |  | 643 | 1 |
| S11 | Guo et al. (2022) | -0.158 [-0.208, -0.107] |  |  | 1433 | 1 |
| S12 | Han et al. (2023) | -0.279 [-0.304, -0.253] |  |  | 4959 | 1 |
| S13 | HariPriya et al. (2019) | -0.335 [-0.489, -0.160] |  |  | 113 | 1 |
| S14 | Huang et al. (2022) | -0.021 [-0.113, 0.071] |  |  | 452 | 1 |
| S15 | Kim & Ahn (2023) | -0.176 [-0.216, -0.136] |  |  | 2242 | 1 |
| S16 | Kim S.-E. et al. (2015) | -0.578 [-0.691, -0.438] |  |  | 110 | 1 |
| S17 | Kumar et al. (2024) | -0.377 [-0.512, -0.224] |  |  | 138 | 1 |
| S18 | Jin C. et al. (2024) | -0.133 [-0.196, -0.069] |  |  | 930 | 1 |
| S19 | Li X.-P. et al. (2023) | -0.220 [-0.301, -0.136] |  |  | 509 | 1 |
| S20 | Lin B. et al. (2022) | -0.153 [-0.198, -0.107] |  |  | 1787 | 1 |
| S21 | Liu & Sun (2023) | -0.210 [-0.293, -0.124] |  |  | 488 | 1 |
| S22 | Lu T. et al. (2022) | -0.058 [-0.078, -0.038] |  |  | 9569 | 1 |
| S23 | Niu S. (2023) | -0.100 [-0.185, -0.014] |  |  | 514 | 1 |
| S24 | Numanoglu-Akbas et al. (2020) | -0.126 [-0.238, -0.011] |  |  | 288 | 1 |
| S25 | Sezer Efe et al. (2023) | -0.107 [-0.199, -0.013] |  |  | 437 | 1 |
| S26 | Shen B. (2024) | -0.233 [-0.297, -0.166] | +0.368 [0.307, 0.426] | -0.320 [-0.381, -0.256] | 802 | 3 |
| S27 | Su et al. (2024) | -0.165 [-0.227, -0.101] |  |  | 913 | 1 |
| S28 | Zhang D. et al. (2022) | -0.223 [-0.295, -0.149] |  |  | 649 | 1 |
| S29 | Tong & Meng (2023) | -0.713 [-0.727, -0.698] |  |  | 4399 | 1 |
| S30 | Tong W.-X. et al. (2022) | -0.173 [-0.204, -0.141] |  |  | 3609 | 1 |
| S31 | Wan & Ren (2023) | -0.278 [-0.356, -0.196] |  |  | 516 | 1 |
| S32 | Wang J. & Hao (2023) | -0.099 [-0.157, -0.040] |  |  | 1112 | 1 |
| S33 | Wang J. et al. (2024) | -0.120 [-0.230, -0.007] |  |  | 301 | 1 |
| S34 | Wei Z. (2023) | -0.270 [-0.326, -0.212] |  |  | 1013 | 1 |
| S35 | Xiao S. (2022) | -0.113 [-0.147, -0.078] |  |  | 3122 | 1 |
| S36 | Xu C.-Y. et al. (2023) | -0.165 [-0.192, -0.138] |  |  | 5075 | 1 |
| S37 | Yang G. et al. (2019) | -0.124 [-0.202, -0.045] |  |  | 608 | 1 |
| S38 | Zeng M. et al. (2022) | -0.140 [-0.184, -0.095] |  |  | 1843 | 1 |
| S39 | Zhang L.-Q. & Gao H.-N. (2023) | -0.747 [-0.781, -0.708] | +0.765 [0.728, 0.797] | -0.786 [-0.816, -0.752] | 560 | 3 |
| S40 | Zhao X. et al. (2024) | -0.140 [-0.181, -0.098] |  |  | 2131 | 1 |
| S41 | Zheng & Ma (2020) | -0.134 [-0.227, -0.039] |  |  | 418 | 1 |
| S42 | Zhu L. et al. (2023) | -0.151 [-0.217, -0.084] |  |  | 823 | 1 |
| S43 | Belaire et al. (2024) |  | +0.294 [0.214, 0.370] |  | 523 | 1 |
| S44 | Cui W. (2022) |  | +0.420 [0.362, 0.475] |  | 824 | 1 |
| S45 | Ho et al. (2015) |  | +0.250 [0.183, 0.315] |  | 775 | 1 |
| S46 | Li H.-Y. et al. (2022) |  | +0.234 [0.175, 0.292] |  | 1009 | 1 |
| S47 | Li et al. (2021) |  | +0.470 [0.425, 0.513] |  | 1214 | 1 |
| S48 | Liu C.-H. (2020) |  | +0.359 [0.313, 0.404] |  | 1408 | 1 |
| S49 | Meng Q.-R. et al. (2024) |  | +0.656 [0.613, 0.695] |  | 746 | 1 |
| S50 | Zhang Z. et al. (2022) |  | +0.098 [0.040, 0.156] |  | 1117 | 1 |
| S51 | Zhang M et al. (2023) |  | +0.249 [0.216, 0.282] |  | 3143 | 1 |
| S52 | Zhao Z. et al. (2022) |  | +0.195 [0.074, 0.310] | -0.250 [-0.361, -0.132] | 257 | 2 |
| S53 | Zhou H. (2022) |  | +0.190 [0.119, 0.259] |  | 722 | 1 |
| S54 | Hu B. et al. (2024) |  |  | -0.099 [-0.126, -0.072] | 5048 | 1 |
| S55 | Xie G. et al. (2023) |  |  | -0.132 [-0.155, -0.109] | 7234 | 1 |
| S56 | Ma A. et al. (2022) |  |  | -0.405 [-0.439, -0.370] | 2268 | 1 |
| S57 | Tian F. et al. (2025) |  |  | -0.327 [-0.412, -0.237] | 401 | 1 |
| S58 | Wu J.-X. et al. (2024) |  |  | -0.376 [-0.411, -0.340] | 2230 | 1 |

**Note on missing-data handling and uncertainty propagation**

Among the 58 studies, only 2 reported all three pairwise correlations, 1 reported two pairs, and the remaining 55 reported a single pair. The unreported correlations were handled by full-information maximum-likelihood (FIML) estimation under a missing-at-random (MAR) assumption, implemented in metaSEM::tssem1. Because the three pairwise correlations were estimated from partly non-overlapping sets of studies, tssem1 jointly estimates the 3×3 pooled correlation matrix and its asymptotic covariance matrix under a random-effects model (REML). In Stage 2, the asymptotic covariance matrix of the Stage-1 pooled correlations was supplied to metaSEM::tssem2 (or an equivalent lavaan specification) as the sampling covariance matrix, so that Stage-1 estimation uncertainty is propagated into the standard errors and bootstrap confidence intervals of the Stage-2 path coefficients and the indirect effect (a × b).

**Part B. Stage-1 pooled correlation vector and asymptotic covariance matrix**

B.1 Stage-1 pooled 3×3 correlation matrix (tssem1, REML):

|  | **PA** | **PR** | **MPA** |
| --- | --- | --- | --- |
| **PA** | 1.000 | 0.370 | −0.225 |
| **PR** | 0.370 | 1.000 | −0.362 |
| **MPA** | −0.225 | −0.362 | 1.000 |

B.2 Asymptotic covariance matrix of the Stage-1 pooled unique correlations (order of elements: PA–MPA, PA–PR, PR–MPA), computed in r-space. The diagonal entries are the squared standard errors of the Stage-1 pooled correlations, obtained from joint multivariate (three-variable) random-effects tssem1 model estimated in Fisher z-space, back-transformed by the delta method.

|  | **PA-MPA** | **PA-PR** | **PR-MPA** |
| --- | --- | --- | --- |
| **PA-MPA** | 0.000876 | 0.000006 | -0.000009 |
| **PA-PR** | 0.000006 | 0.003411 | 0.000040 |
| **PR-MPA** | -0.000009 | 0.000040 | 0.007255 |

**Part C. Stage-2 model specification**

The Stage-2 model was specified as a mediation path model with psychological resilience (PR) mediating the relationship between physical activity (PA) and mobile phone addiction (MPA): PA → PR → MPA, with a direct PA → MPA path. The pooled Stage-1 correlation matrix (Part B.1) was supplied as the target and its asymptotic covariance matrix (Part B.2) as the sampling covariance matrix. The estimated path coefficients and effects are summarized below.

| **Path** | **Label** | **Estimate** |
| --- | --- | --- |
| PA → PR | a | 0.370 |
| PR → MPA | b | −0.324 |
| PA → MPA (direct) | c′ | −0.105 |
| Indirect effect (a × b) | — | −0.120 |
| Total effect (c′ + a × b) | — | −0.225 |
| Proportion mediated | — | 53.3% |

\# Stage 1: pooled correlation matrix and its asymptotic covariance matrix
library(metaSEM)
tssem1.obj <- tssem1(Cov = Corlist, # list of study-level 3x3 correlation matrices
 n = Nlist, # list of study-level sample sizes
 method = "REML",
 RE.type = "Symm")
summary(tssem1.obj)	# pooled vector + acov (Part B)

\# Stage 2: MASEM with pooled matrix as the target and acov as sampling covariance
tssem2.obj <- tssem2(tssem1.obj,
 model = "PA -> PR -> MPA; PA -> MPA",
 RE.type = "Symm")
summary(tssem2.obj)

\# Equivalent lavaan syntax (for transparency)
model <- '
 PR ~ a*PA	# a = 0.370
 MPA ~ b*PR + cp*PA # b = -0.324, cp = -0.105
 ab := a*b	# indirect effect = -0.120
 total := cp + a*b	# total effect	= -0.225
'
\# Indirect effect (a*b) accounted for 53.3% of the total PA->MPA effect.

**Part D. Convergence information**

Both stages were estimated with the default optimizer in metaSEM; convergence was successful (status 0). Stage 2 is a saturated model (df = 0), so RMSEA/CFI/TLI are 0/1 by construction and are not interpreted as evidence of model fit.

| **Convergence criterion** | **Result** |
| --- | --- |
| Optimizer | NLMINB (metaSEM default) |
| Stage-1 tssem1 status | 0 (converged) |
| Stage-1 method | REML; random-effects variance estimated per pair |
| Stage-2 tssem2 model | saturated / just-identified (df = 0) |
| RMSEA / CFI / TLI | 0 / 1 / 1 by construction (not reported as fit evidence) |

**Part E. Bootstrap procedure**

A case/study bootstrap with 5,000 resamples was used to obtain the 95% confidence interval of the indirect effect. For each resample, Stage-1 and Stage-2 were re-estimated and the indirect effect (a × b) recorded; the 95% CI was taken from the 2.5th and 97.5th percentiles of the 5,000 bootstrap estimates.

| **Bootstrap parameter** | **Value** |
| --- | --- |
| Resampling method | case / study bootstrap |
| Number of resamples | 5,000 |
| Re-estimated per sample | Stage-1 + Stage-2 |
| Indirect effect (a × b) | −0.120 |
| 95% CI | [−0.209, −0.039] |
| Significance | 95% CI excludes 0 |

**Publication bias: Funnel plots**

**Supplementary Figure S1：Funnel Plot** **for Physical Activity & Mobile Phone Addiction**

**Supplementary Figure S2: Funnel Plot for Psychological Resilience & Mobile Phone Addiction**

**Supplementary Figure S3: Funnel Plot for Physical Activity & Psychological Resilience**

**The leave-one-out sensitivity analysis results**

**Supplementary Figure S**4: Sensitivity Analysis Plot for Physical Activity & Mobile Phone Addiction

**Supplementary Figure S5**: Sensitivity Analysis Plot for Psychological Resilience & Mobile Phone Addiction

**Supplementary Figure S6**: Sensitivity Analysis Plot for Physical Activity & Psychological Resilience
